# Co_3_O_4_/NiO@GQD@SO_3_H nanocomposite as a superior catalyst for the synthesis of chromenpyrimidines†

**DOI:** 10.1039/c9ra05896f

**Published:** 2019-11-15

**Authors:** Javad Safaei-Ghomi, Zahra Omidshafiei

**Affiliations:** Department of Organic Chemistry, Faculty of Chemistry, University of Kashan Kashan P. O. Box 87317-51167 I. R. Iran safaei@kashanu.ac.ir +98-31-55912397 +98-31-55912385

## Abstract

A three-component reaction involving aromatic aldehydes, 6-amino-1,3-dimethyluracil and 4-hydroxycoumarin was achieved in the presence of the Co_3_O_4_/NiO@GQD@SO_3_H nanocomposite as a highly effective heterogeneous catalyst to produce chromenpyrimidines. The catalyst was characterized *via* FT-IR, SEM, XRD, EDS, TGA, BET and VSM. This new catalyst was demonstrated to be highly effective in the preparation of chromenpyrimidines. Atom economy, low catalyst loading, reusable catalyst, applicability to a wide range of substrates and high product yields are some of the important features of this protocol.

## Abstract 2

A flexible and highly efficient protocol for the synthesis of chromenpyrimidines using the Co_3_O_4_/NiO@GQD@SO_3_H nanocomposite has been developed.

## Graphical abstract

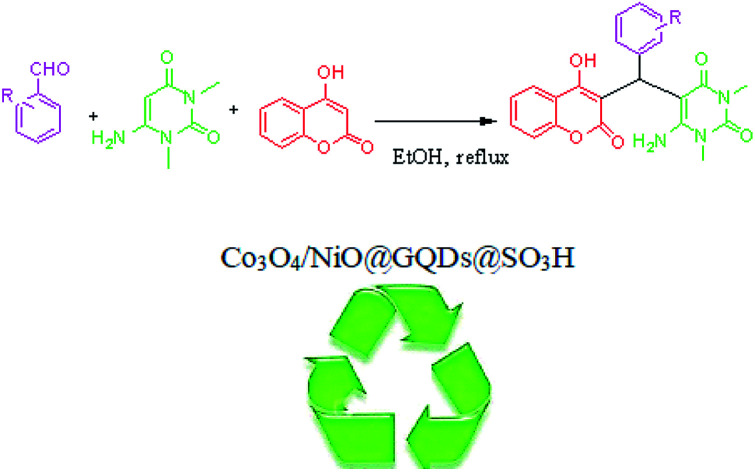

## 1. Introduction

**Scheme 1:**
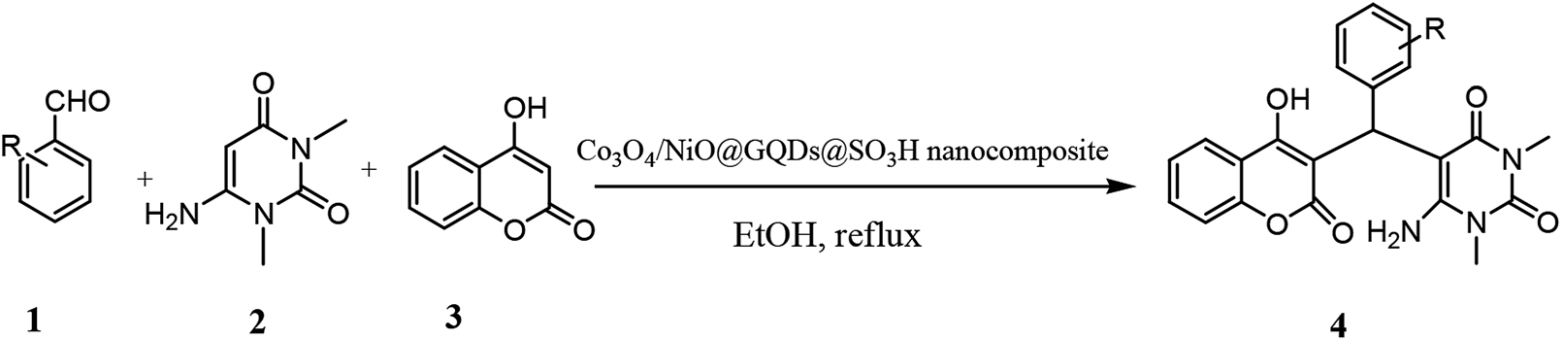
Synthesis of chromenpyrimidines using the Co_3_O_4_/NiO@GQD@SO_3_H nanocomposite.

Chromenpyrimidines are a common scaffold in multiple bioactive compounds and possess several pharmacological properties.^1^ These compounds are used as analgesic, anti-pyretic,^2^ anti-microbial,^3,4^ anti-biofilm,^5^ anti-inflammatory,^6^ anticancer,^7^ antitubercular agents.^8^ Some other examples of pyrimidines as prominent drug molecules include uramustine, piritrexim isetionate, tegafur, floxuridine, methotrexate, trimethoprim, piromidic acid, tetroxoprim and dipyridamole, which have high bioavailability, slow onset and prolonged effect.^9^ Chromenpyrimidines have been regarded as significant targets of organic synthesis. Therefore, the development of effective methods for the preparation of chromenpyrimidines is an attractive challenge. Several methods have been reported for the preparation of chromenpyrimidines in the presence of diverse catalysts including l-proline-derived secondary aminothiourea,^10^ sulfamic acid,^11^ Zr(HSO_4_)_4_,^12^l-proline,^13^ and bifunctional thiourea-based organocatalyst.^14^ However, some of the reported procedures have disadvantages including long reaction times, use of toxic and non-reusable catalysts and undesirable reaction conditions. Therefore, to avoid these drawbacks, the search for effective methods for the preparation of chromenpyrimidines is still desirable. Nanoparticles exhibit good catalytic activity owing to their large surface area and active sites. Metal oxides are a broad class of materials that have been researched extensively due to their unique attributes and potential applications in various fields. Graphene quantum dots (GQDs) are a new member of the carbon nanostructure family, which have quasi-spherical structures. GQDs have gained intensive attention due to their significant features, biological,^15^ biomedical,^16^ and therapeutic applications,^17^ as a new class of photocatalysts^18^ and surfactants,^19^ and electrochemical biosensing,^20^ electrocatalytic,^21^ lithium battery,^22^ optical and photovoltaic,^23^ photoluminescence,^24,25^ bioimaging,^26^ and catalytic applications.^27^ The potential applications of N-graphene quantum dots were recently reviewed based on experimental and theoretical studies.^28–31^ The synthesis of highly efficient nanocomposite catalysts for the synthesis of organic compounds is still a big challenge. To obtain a larger surface area and more active sites, nanocatalysts are functionalized with active groups.^32–34^ It has been demonstrated that the decoration of nanocatalysts with GQDs prevents the aggregation of fine particles, and thus increases the effective surface area and number of reactive sites for efficient catalytic reactions. The chemical groups on GQD can catalyze chemical reactions, and their –COOH and –SO_3_H groups can serve as acid catalysts for many reactions.^27–36^ Herein, we report the use of a Co_3_O_4_/NiO@GQD@SO_3_H nanocomposite as a new efficient catalyst for the preparation of chromenpyrimidines through a three-component reaction involving aromatic aldehydes, 6-amino-1,3-dimethyluracil and 4-hydroxycoumarin (Scheme 1).

## 2. Results and discussion

**Fig. 1:**
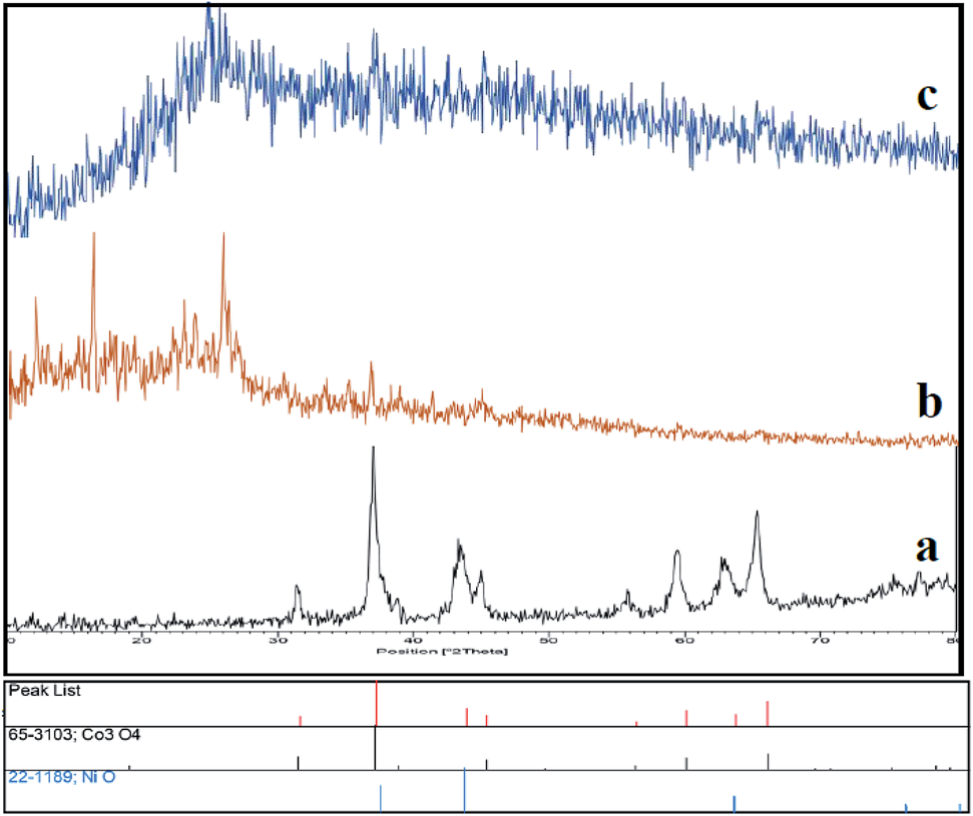
XRD patterns of (a) Co_3_O_4_/NiO, (b) Co_3_O_4_/NiO@GQDs and (c) Co_3_O_4_/NiO@GQD@SO_3_H.

Initially, we prepared Co_3_O_4_/NiO nanoparticles *via* simple techniques. A facile hydrothermal method was used for the preparation of N-GQDs.^37^ Sulfonated graphene quantum dots were prepared using chlorosulfonic acid.^38^ The XRD patterns of Co_3_O_4_/NiO, Co_3_O_4_/NiO@N-GQDs and Co_3_O_4_/NiO@GQD@SO_3_H nanocomposite are shown in Fig. 1, which confirm the presence of both NiO (JCPDS No.22-1189) and Co_3_O_4_ (JCPDS no 65-3103).

**Fig. 2:**
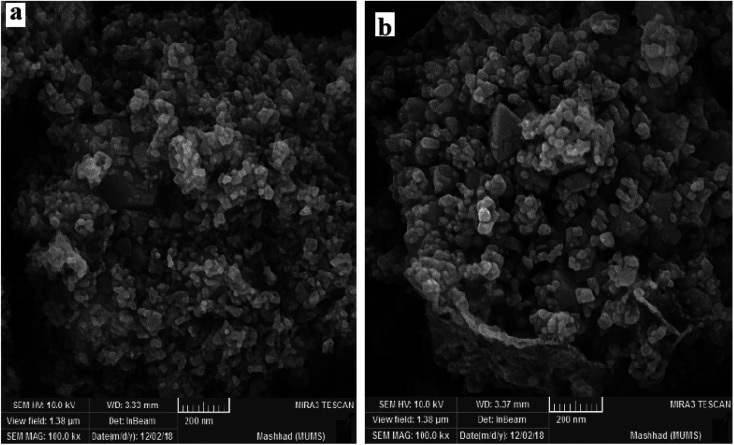
SEM images of (a) Co_3_O_4_/NiO and (b) Co_3_O_4_/NiO@GQD@SO_3_H.

**Fig. 3:**
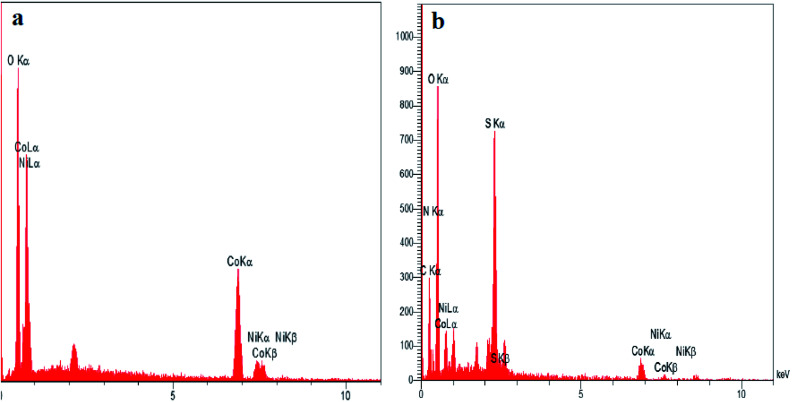
EDS spectra of (a) Co_3_O_4_/NiO and (b) Co_3_O_4_/NiO@GQD@SO_3_H.

To investigate the morphology and particle size of the nanoparticles, SEM images of Co_3_O_4_/NiO and Co_3_O_4_/NiO@ GQD@SO_3_H nanocomposite were measured, as shown in Fig. 2. The SEM images of the Co_3_O_4_/NiO@GQD@SO_3_H nanocomposite show the formation of uniform particles, and the energy-dispersive X-ray spectrum (EDS) confirms the presence of Co, Ni, O, S and C species in the structure of the nanocomposite (Fig. 3).

**Fig. 4:**
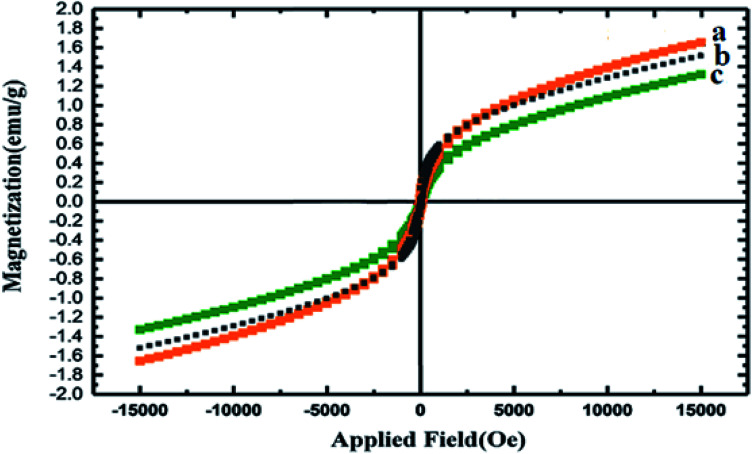
VSM curves of (a) Co_3_O_4_/NiO, (b) Co_3_O_4_/NiO@GQDs and (c) Co_3_O_4_/NiO@GQD@SO_3_H.

The magnetic properties of the nanocomposites before and after their decoration with GQDs were tested using a vibrating-sample magnetometer (VSM) (Fig. 4). The lower magnetism of the as-synthesized Co_3_O_4_/NiO@GQD@SO_3_H compared with that of Co_3_O_4_/NiO is ascribed to the antiferromagnetic behavior of the GQDs as a dopant. These results demonstrate that the magnetization property decreased by coating and functionalization.^39,40^

**Fig. 5:**
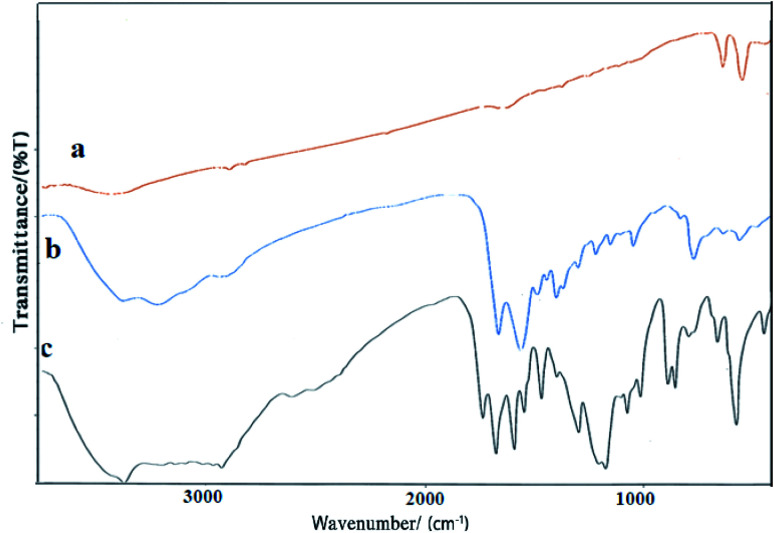
FT-IR spectra of (a) Co_3_O_4_/NiO, (b) Co_3_O_4_/NiO@GQDs and (c) Co_3_O_4_/NiO@GQD@SO_3_H.

The FT-IR spectra of Co_3_O_4_/NiO, Co_3_O_4_/NiO@N-GQD and Co_3_O_4_/NiO@GQD@SO_3_H nanocomposite are shown in Fig. 5. The absorption peak at 3335 cm^−1^ is related to the stretching vibrational absorptions of OH. The peaks at 461.4, 568.4, and 657.1 cm^−1^ correspond to Ni–O, Co^2+^–O and Co^3+^–O respectively. The characteristic peaks at 3440 cm^−1^ (O–H stretching vibration), 1705 cm^−1^ (C=O stretching vibration), and 1125 cm^−1^ (C–O–C stretching vibration) appear in the spectrum in Fig. 5b. The peak at approximately 1475–1580 cm^−1^ is attributed to the C=C bonds. The presence of the sulfonyl group is also verified by the peaks at 1215 and 1120 cm^−1^. The broad peak at 3350 cm^−1^ is related to the stretching vibrational absorptions of OH (SO_3_H) (Fig. 5c).

**Fig. 6:**
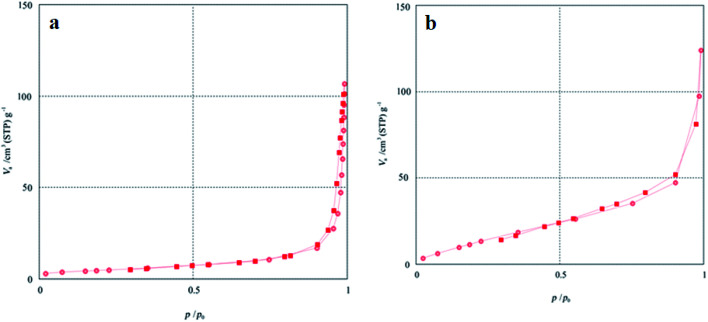
BET specific surface area of (a) Co_3_O_4_/NiO and (b) Co_3_O_4_/NiO@GQD@SO_3_H.

The BET specific surface area of the Co_3_O_4_/NiO and Co_3_O_4_/NiO@GQD@SO_3_H nanocomposites was measured by nitrogen gas adsorption–desorption isotherms (Fig. 6). The results indicate that the BET specific surface area of Co_3_O_4_/NiO improved from 12.25 to 32.43 m^2^ g^−1^ after modification with the GQDs; therefore, more active sites were introduced on the surface of Co_3_O_4_/NiO@GQD@SO_3_H.

**Fig. 7:**
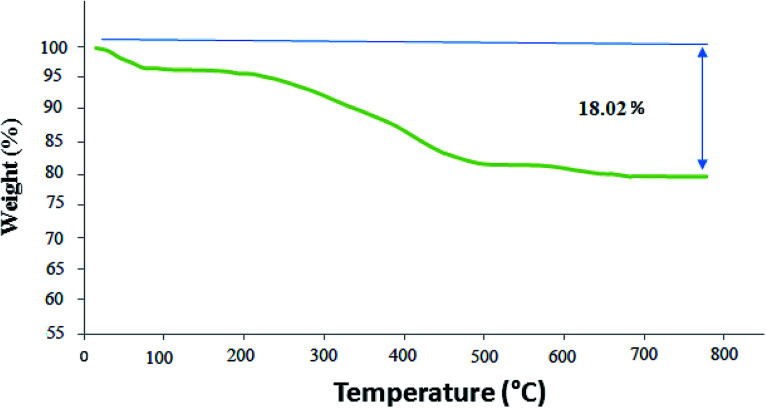
TGA of Co_3_O_4_/NiO@GQD@SO_3_H nanocomposite.

TGA (thermogravimetric analysis) was used to evaluate the thermal stability of the Co_3_O_4_/NiO@GQD@SO_3_H nanocomposite. The nanocomposite displayed suitable thermal stability without a significant decrease in weight (Fig. 7). The weight loss at temperatures below 210 °C is owing to the removal of physically adsorbed solvent and surface hydroxyl groups. The curve indicates a weight loss of about 14.06% from 150 °C to 500 °C, which is attributed to the oxidation and degradation of the GQD.

**Fig. 8:**
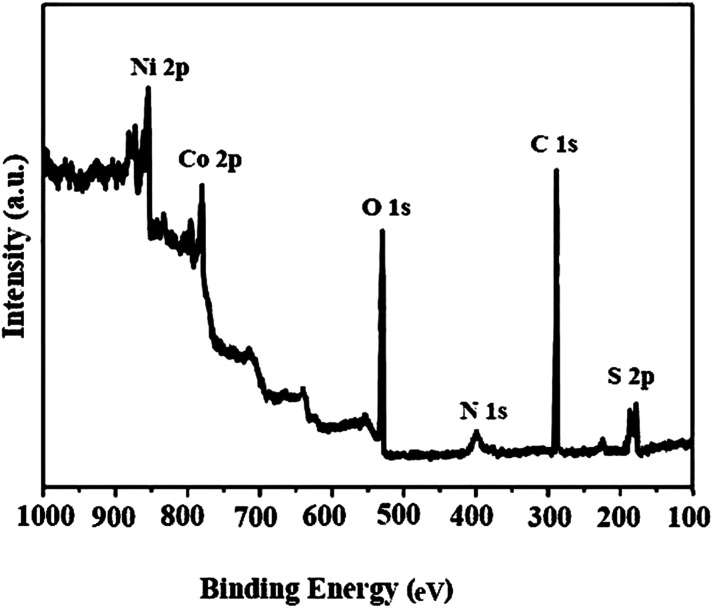
X-ray photoelectron spectroscopy (XPS) analysis of the Co_3_O_4_/NiO@GQD@SO_3_H nanocomposite.

The X-ray photoelectron spectroscopy (XPS) analysis of the Co_3_O_4_/NiO@GQD@SO_3_H nanocomposite is shown in Fig. 8. In the wide-scan spectrum of the nanocatalyst, the predominant components are Ni 2p_1/2_ (873.4 eV), Ni 2p_3/2_ (854.4 eV), Co 2p_1/2_ (792.6 eV), Co 2p_3/2_ (780.4 eV), O 1s (529.8 eV), N 1s (400 eV), C 1s (284.5 eV) and S 2p (164.3 eV).

Initially, we carried out a three-component reaction with 4-nitrobenzaldehyde, 6-amino-1,3-dimethyluracil and 4-hydroxycoumarin as a model reaction. The model reactions were performed in the presence of NaHSO_4_, ZrO_2_, *p*-TSA, Co_3_O_4_, NiO, Co_3_O_4_/NiO, Co_3_O_4_/NiO@GQDs and Co_3_O_4_/NiO@GQD@SO_3_H nanocomposite as catalysts. The reactions were tested using diverse solvents including ethanol, acetonitrile, water and dimethylformamide. The best results were obtained in EtOH and we found that the reaction gave convincing results in the presence of the Co_3_O_4_/NiO@GQD@SO_3_H nanocomposite (5 mg) under reflux conditions (Table 1).

**Table 1:** Optimization of the reaction conditions using different catalysts^a^

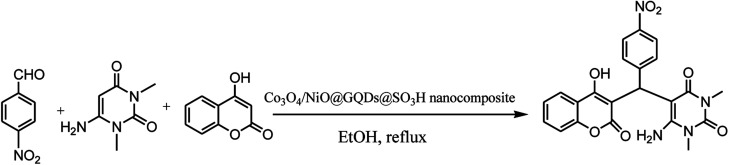					
Entry	Catalyst (amount)	Solvent (reflux)	Time (min)	Conversion efficiency	Yield^b^ (%)
1	None	EtOH	300	NR	NR
2	Et_3_N (5 mol%)	EtOH	300	28	35
3	NaHSO_4_ (4 mol%)	EtOH	250	37	42
4	ZrO_2_ (4 mol%)	EtOH	250	43	48
5	*p*TSA NPs (5 mol%)	EtOH	250	49	54
6	Nano-Co_3_O_4_	EtOH	250	40	45
7	Nano-NiO	EtOH	250	45	52
8	Co_3_O_4_/NiO nanocomposite	EtOH	250	55	60
9	Co_3_O_4_/NiO@GQD nanocomposite	EtOH	150	68	72
10	Co_3_O_4_/NiO@GQD@SO_3_H nanocomposite (3 mg)	EtOH	80	81	85
11	Co_3_O_4_/NiO@GQD@SO_3_H nanocomposite (5 mg)	EtOH	80	87	93
12	Co_3_O_4_/NiO@GQD@SO_3_H nanocomposite (7 mg)	EtOH	80	87	93
13	Co_3_O_4_/NiO@GQD@SO_3_H nanocomposite (5 mg)	H_2_O	100	69	72
14	Co_3_O_4_/NiO@GQD@SO_3_H nanocomposite (5 mg)	DMF	90	71	76
15	Co_3_O_4_/NiO@GQD@SO_3_H nanocomposite (5 mg)	CH_3_CN	80	77	82

a 4-Nitrobenzaldehyde (1 mmol), 6-amino-1,3-dimethyluracil (1 mmol) and 4-hydroxycoumarin (1 mmol).

b Isolated yield.

A series of aromatic aldehydes were studied under the optimum conditions (Table 2). Good yields were obtained using aromatic aldehydes either bearing electron-withdrawing substituents or electron-donating substituents.

**Table 2:** Synthesis of chromenpyrimidines using the Co_3_O_4_/NiO@GQD@SO_3_H nanocomposite (5 mg) under reflux conditions^a^

Entry	Aldehyde (1a–1l)	Product	Time (min)	Conversion efficiency	Yield^b^ (%)	mp (°C) (ref.)	mp (°C) (reported)
1	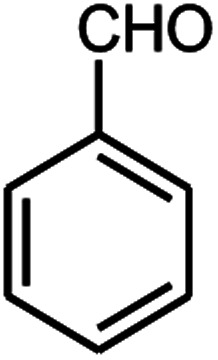	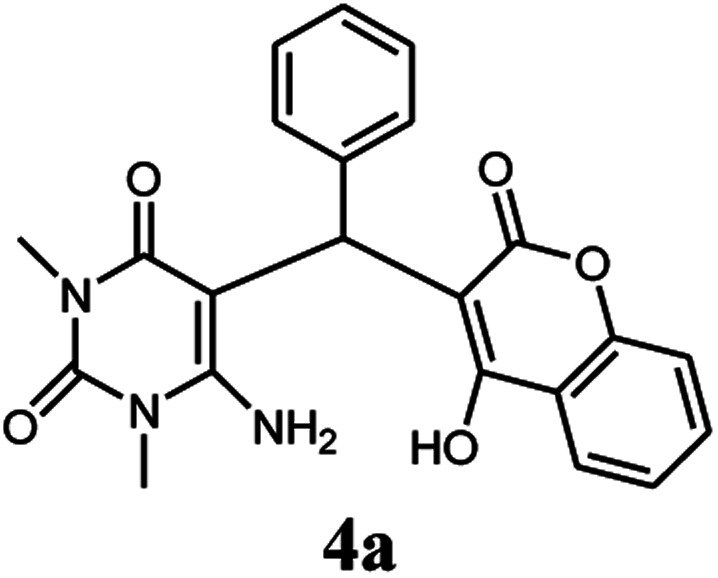	80	80	88	218–220 (10)	220–222
2	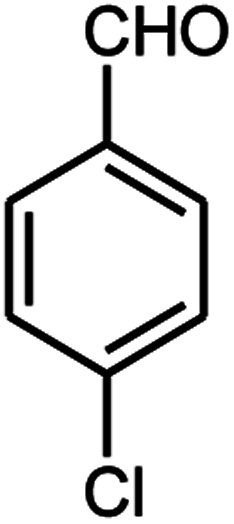	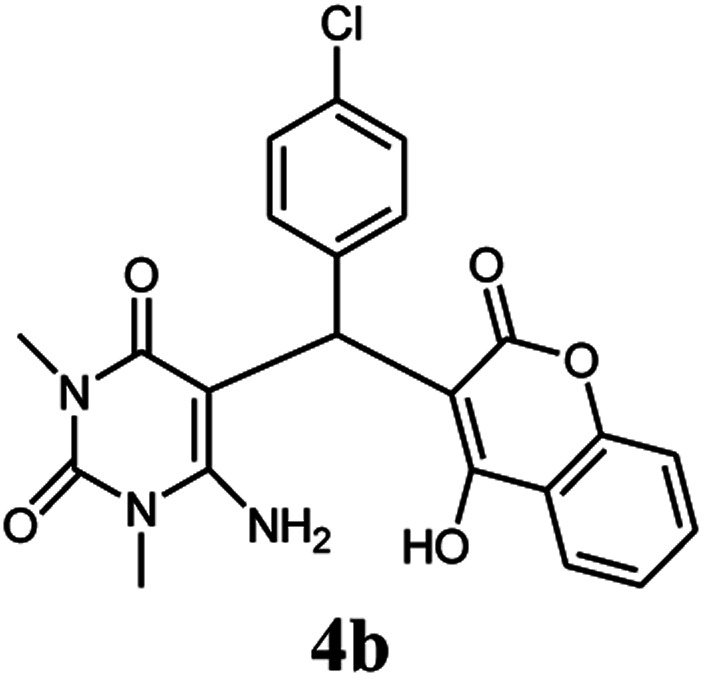	80	82	90	219–220 (10)	218–220
3	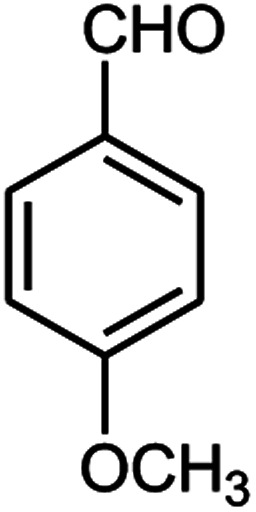	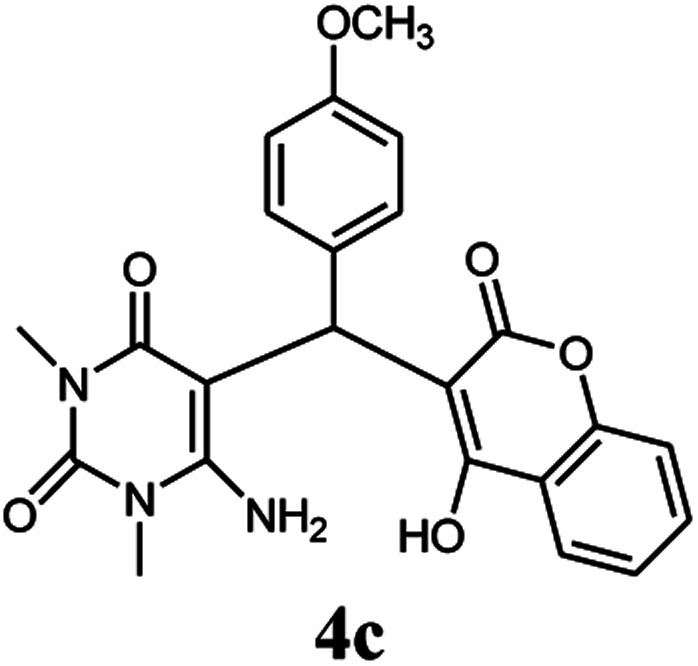	90	72	85	180–181 (10)	178–180
4	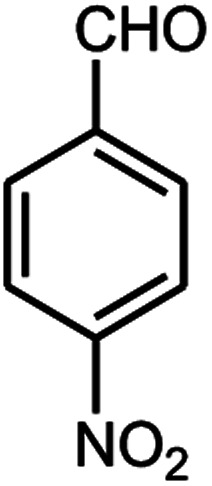	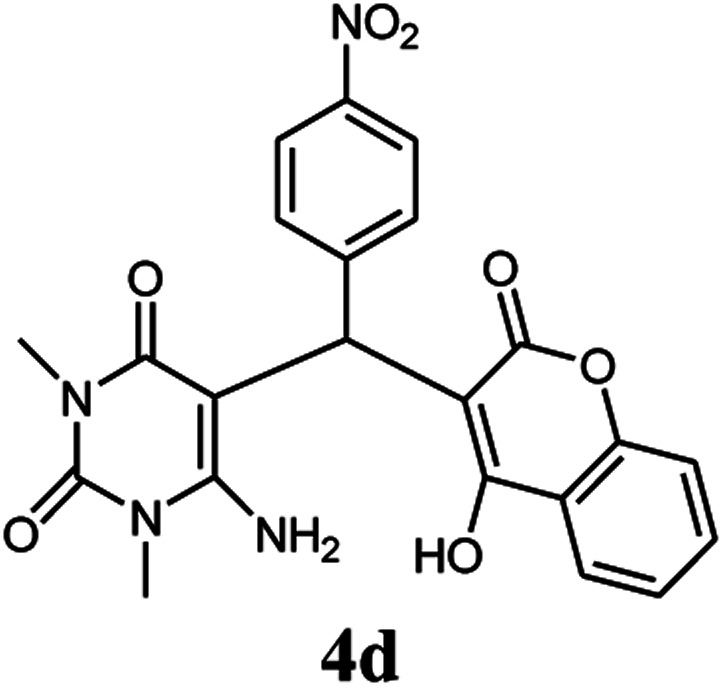	80	87	93	240–242 (13)	240–242
5	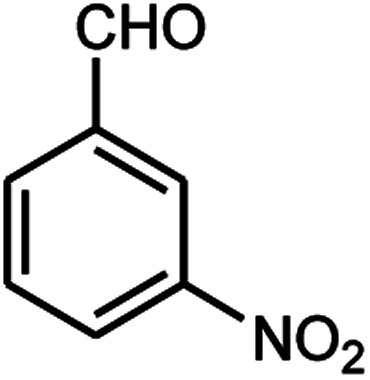	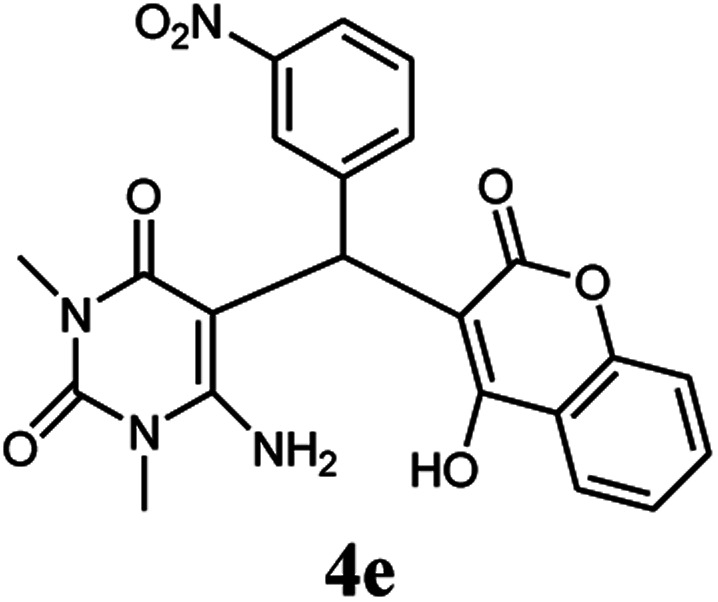	80	80	88	230–232 (10)	230–232
6	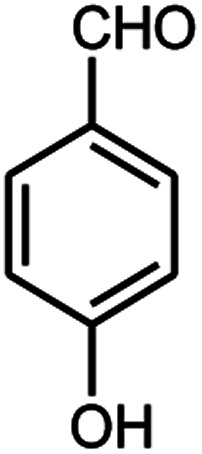	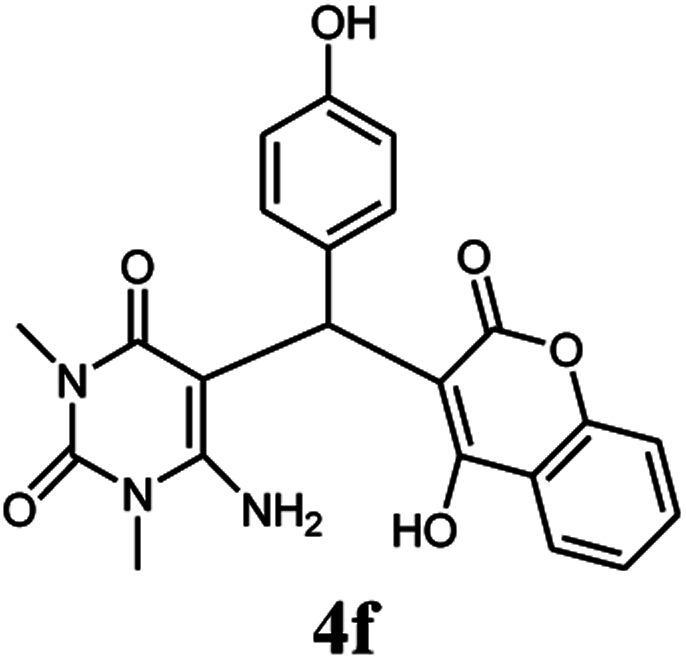	110	75	83	—	230–232
7	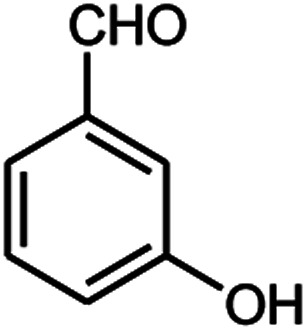	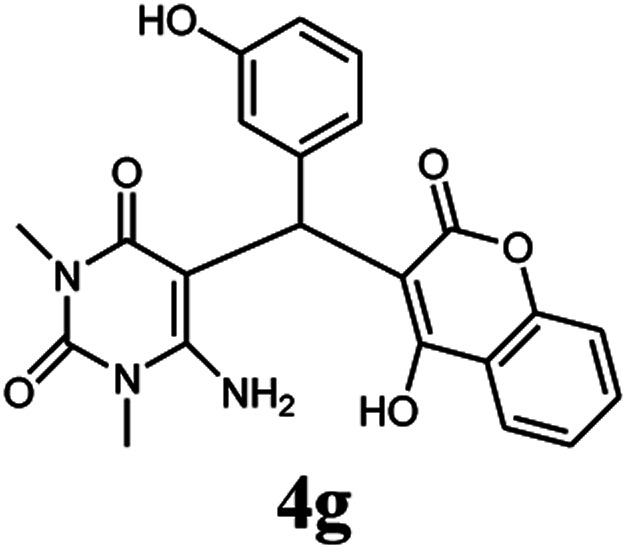	100	77	87	—	232–234
8	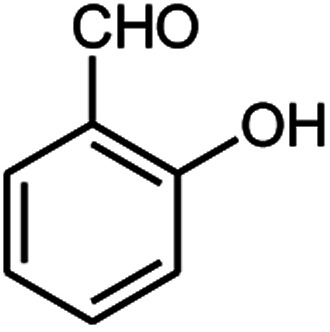	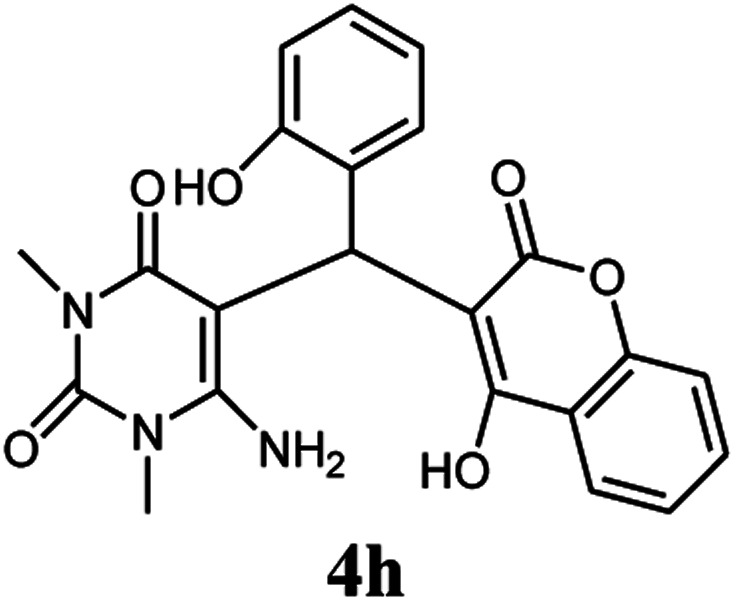	110	74	81	—	240–242
9	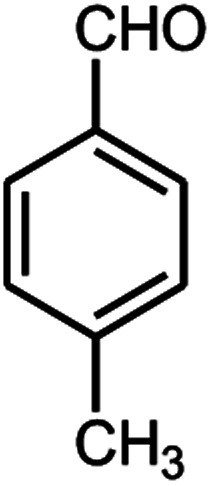	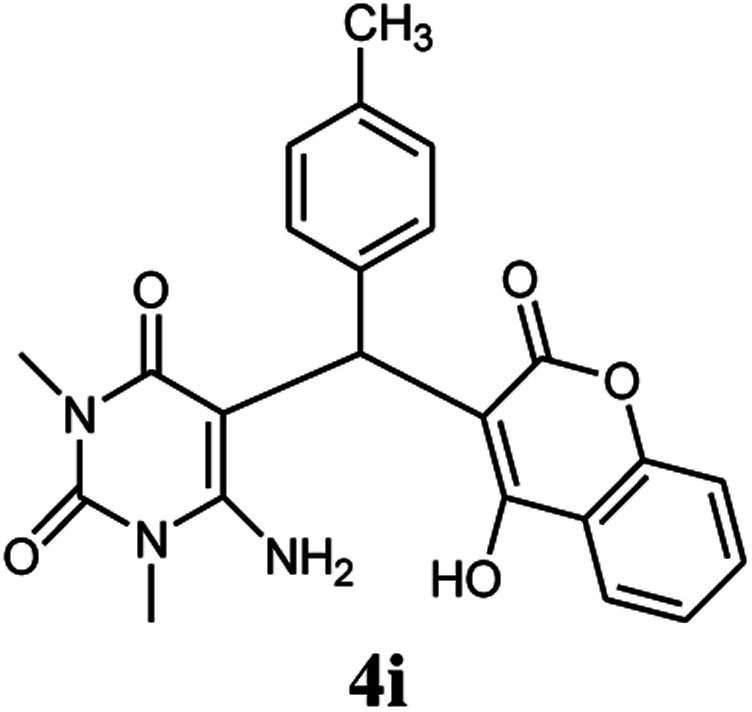	90	76	85	202–204 (10)	202–204
10	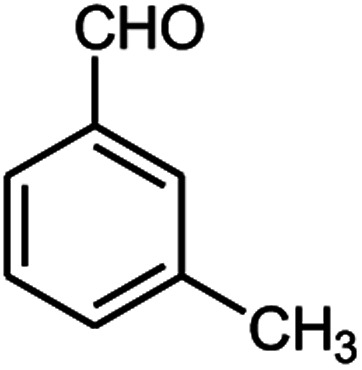	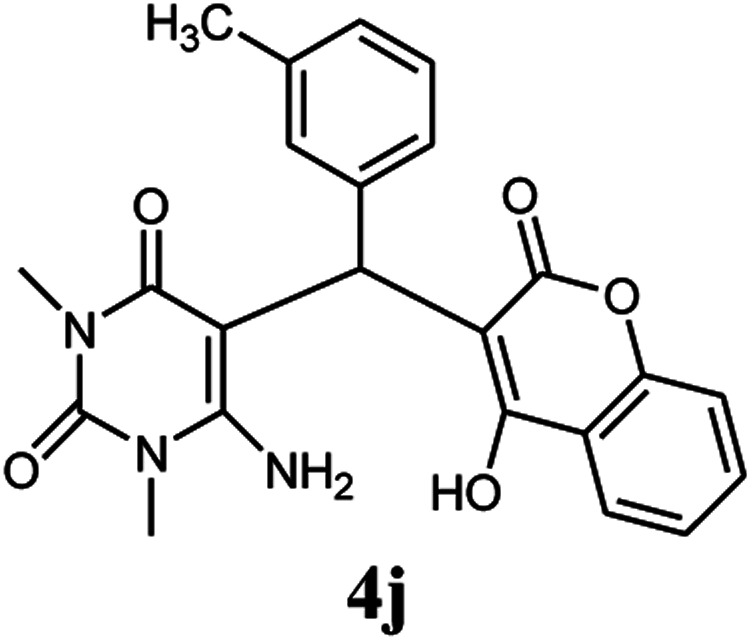	85	80	87	—	200–202
11	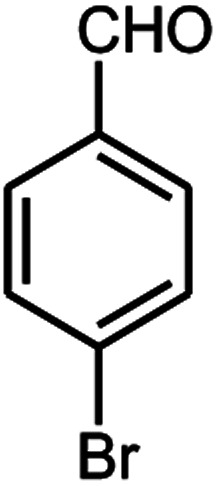	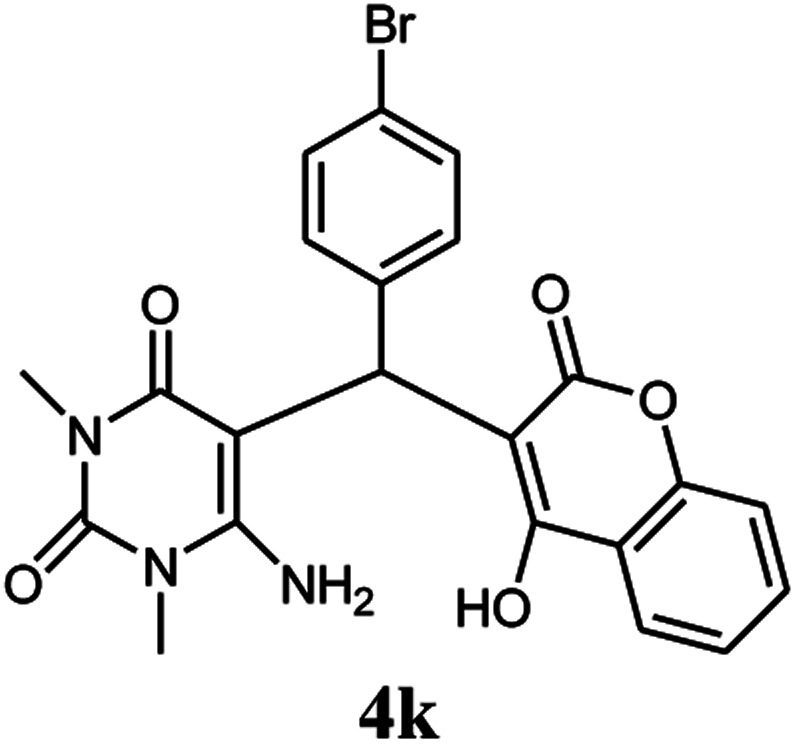	80	81	89	236–238 (10)	236–238
12	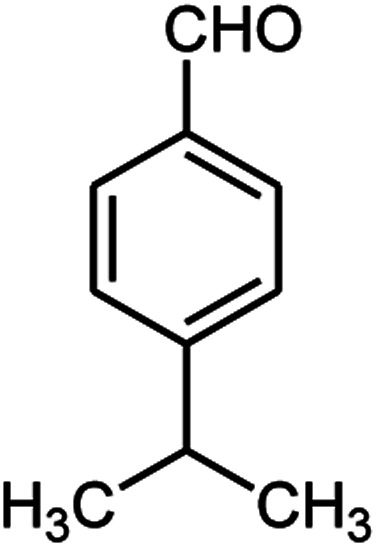	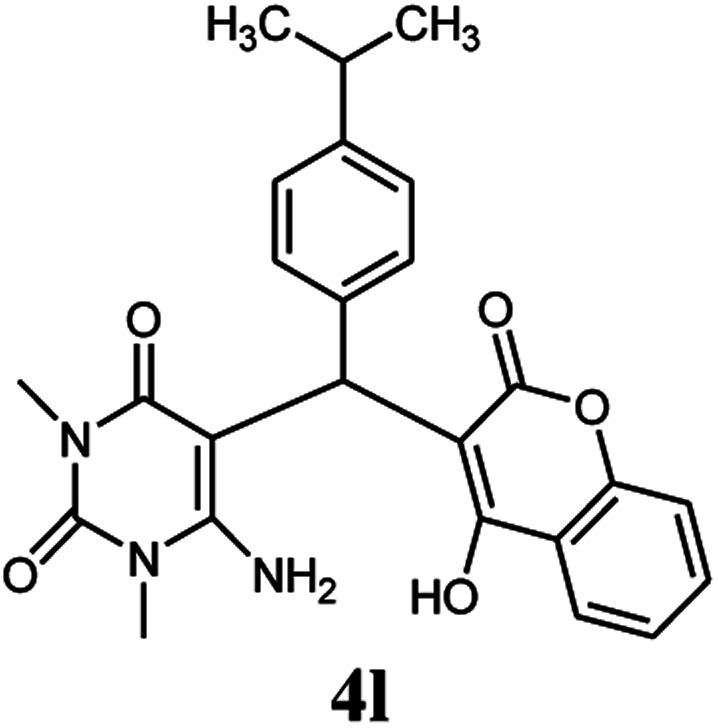	100	74	84	257–259 (13)	257–259
13	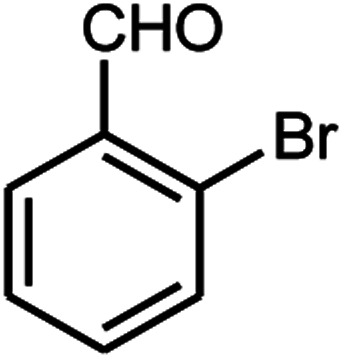	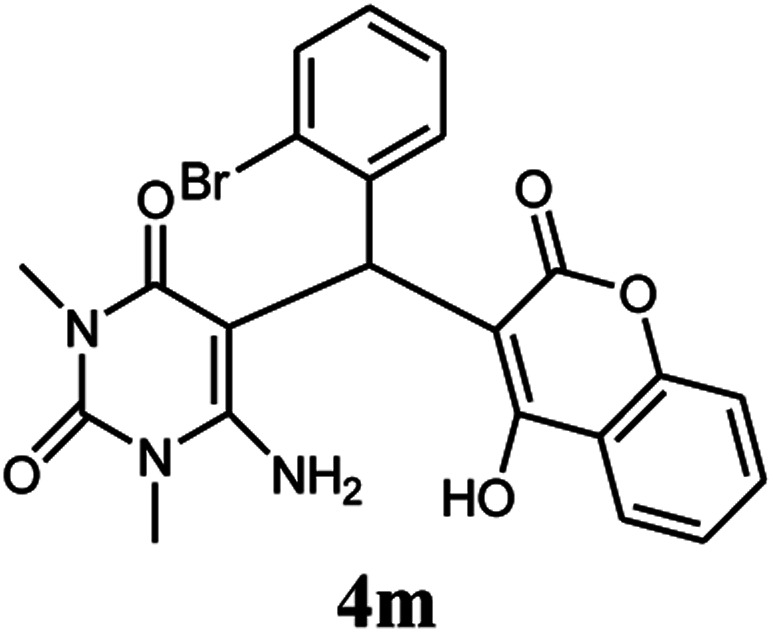	80	79	89	210–212 (10)	210–212
14	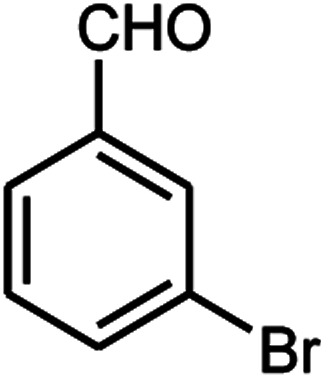	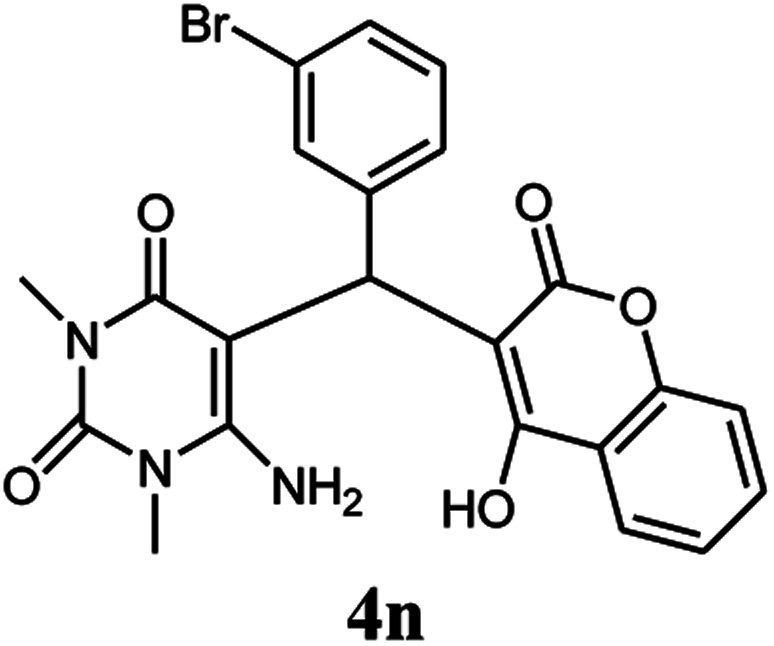	80	81	88	245–247 (13)	245–247

a Aromatic aldehydes (1 mmol), 6-amino-1,3-dimethyluracil (1 mmol) and 4-hydroxycoumarin (1 mmol).

b Isolated yield.

**Fig. 9:**
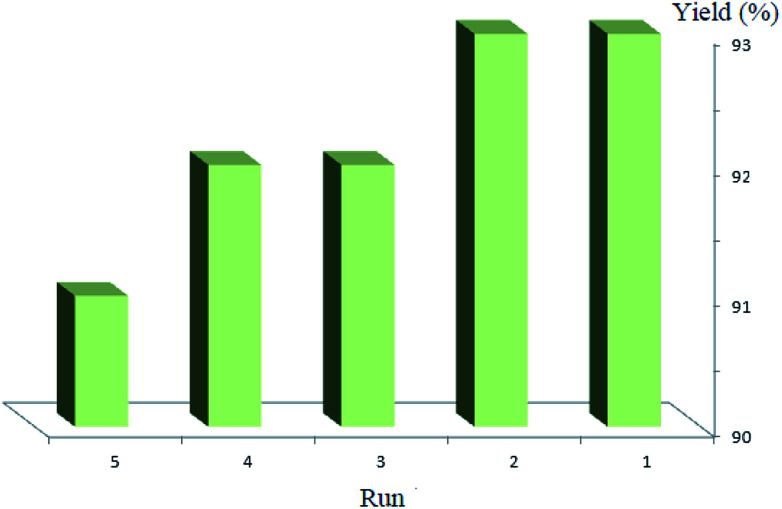
Recycling of the Co_3_O_4_/NiO@GQD@SO_3_H nanocomposite as a catalyst for the model reaction.

We also investigated the recyclability of the Co_3_O_4_/NiO@GQD@SO_3_H nanocomposite as a catalyst for the model reaction under reflux conditions in ethanol. The results showed that nanocomposite can be reused several times without noticeable loss in its catalytic activity (yield in the range of 93% to 91%) (Fig. 9).

**Scheme 2:**
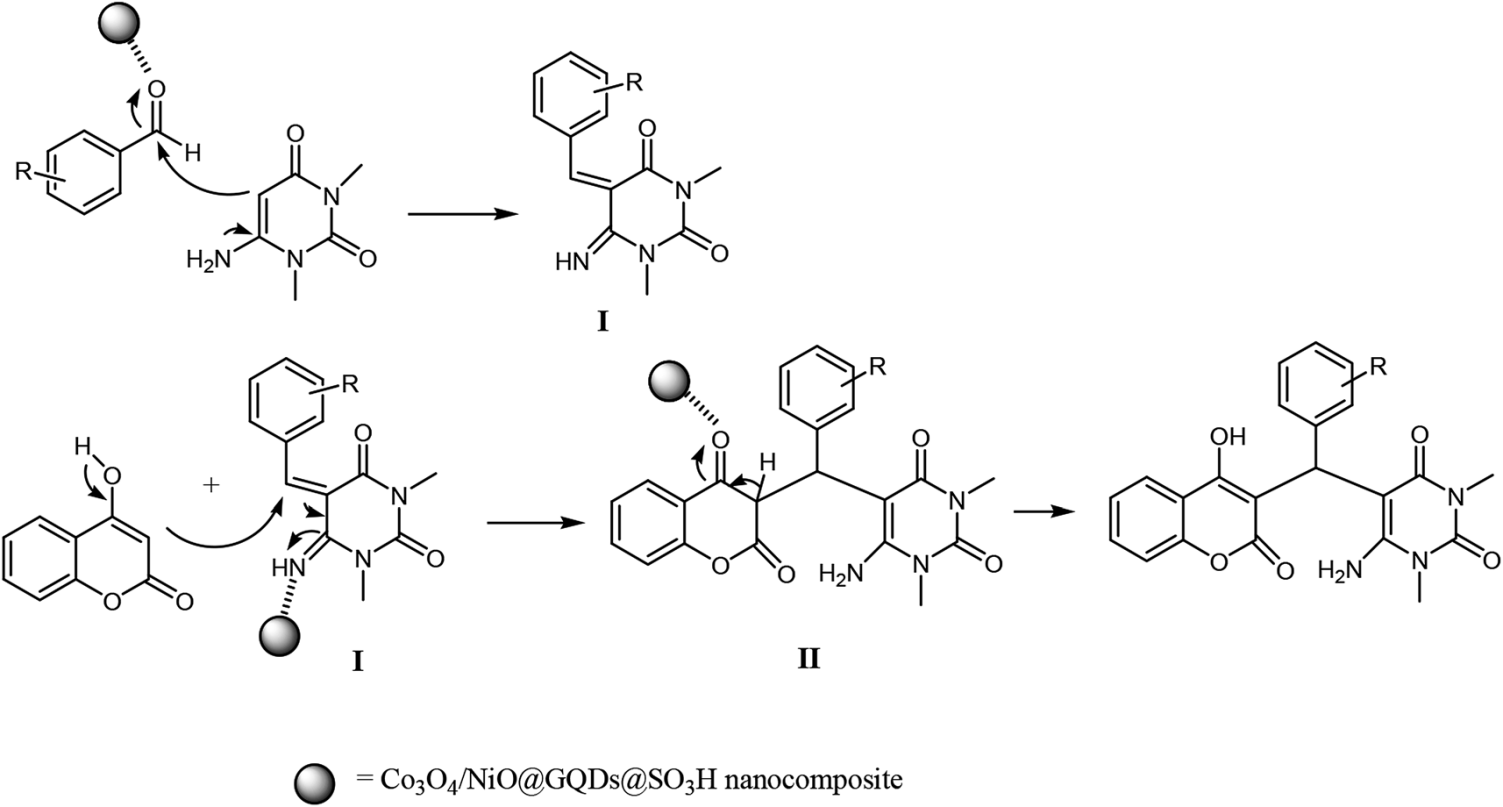
Proposed mechanism for the three-component reaction.

The plausible mechanism for the preparation of chromenpyrimidines using the Co_3_O_4_/NiO@GQD@SO_3_H nanocomposite is shown in Scheme 2. Firstly, we assumed that the reaction occurs *via* condensation between 6-amino-1,3-dimethyluracil and aldehyde to form intermediate I on the active sites of the Co_3_O_4_/NiO@GQD@SO_3_H nanocatalyst. Then, 4-hydroxycoumarin is added to intermediate I to give intermediate II. Then, migration of the hydrogen atom provides the final product (Scheme 2).

To study the applicability of this method for large-scale synthesis, we performed selected reactions at the 10 mmol scale. As can be seen, the reactions on a large scale gave the product with a gradual decrease in reaction yield (Table 3).

**Table 3:** The large-scale synthesis of some chromenpyrimidines using the Co_3_O_4_/NiO@GQD@SO_3_H nanocomposite (5 mg)

Entry	Product	Time (min)	Yield^a^ (%)
1	4a	90	86
2	4c	95	83
3	4d	80	90
4	4i	95	82
5	4k	80	87

a Isolated yield.

To compare the efficiency of the Co_3_O_4_/NiO@GQD@SO_3_H nanocomposite with the reported catalysts for the synthesis of chromenpyrimidines, we tabulated the results in Table 4. As indicated in Table 4, the Co_3_O_4_/NiO@GQD@SO_3_H nanocomposite is superior to the reported catalysts. As expected, the increased surface area due to the small particle size increased the reactivity of the catalyst, which is responsible for the accessibility of the substrate molecules on the catalyst surface.

**Table 4:** Comparison of the catalytic activity of the Co_3_O_4_/NiO@GQD@SO_3_H nanocomposite (5 mg) with that of other reported catalysts for the synthesis chromenpyrimidines (4b)

Entry	Catalyst (conditions)	Time (min)	Conversion efficiency	Yield^a^ (%)	Ref.
1	l-proline, 20 mol%, EtOH, reflux	360	75	83	13
2	Bifunctional thiourea-based organocatalyst, 20 mol%, H_2_O, reflux	240	79	86	14
3	Sulfamic acid, 30 mol%, (EtOH : H_2_O: 1 : 1), ultrasound irradiation	30	77	85	11
4	Co_3_O_4_/NiO@GQD@SO_3_H nanocomposite (5 mg, EtOH, reflux)	80	82	90	This work

a Isolated yield.

The activity of catalysts is influenced by their acid–base properties and many other factors such as surface area, geometric structure (particularly pore structure), distribution of sites and polarity of the surface sites.^41,42^ The SO_3_H groups distributed on the surface of Co_3_O_4_/NiO@GQDs activate the groups of the substrates. In this mechanism, the surface atoms of Co_3_O_4_/NiO@GQD@SO_3_H activate the C=O and C=N groups for better reaction with nucleophiles. The Co_3_O_4_/NiO@GQD@SO_3_H nanocomposite has Lewis and Brønsted acid properties, which increase the activity of the catalyst.

## 3. Experimental

### 3.1. Chemicals and apparatus

NMR spectra were recorded on a Bruker Avance-400 MHz spectrometer in the presence of tetramethylsilane as an internal standard. IR spectra were recorded on an FT-IR Magna 550 spectrometer using KBr plates. Melting points were determined on an Electrothermal 9200, and are not corrected. Elemental analyses (C, H, and N) were performed on a Carlo ERBA Model EA 1108 analyzer. Powder X-ray diffraction (XRD) was performed on a Philips X'pert diffractometer with monochromatized Cu Kα radiation (*λ* = 1.5406 Å). The microscopic morphology of the nanocatalyst was visualized by SEM (MIRA3). Thermogravimetric analysis (TGA) curves were measured on a V5.1A DUPONT 2000. The magnetic measurement of the samples was performed in a vibrating sample magnetometer (VSM) (Meghnatis Daghigh Kavir Co.; Kashan Kavir; Iran).

### 3.2. Preparation of Co_3_O_4_/NiO nanoparticles

Co(NO_3_)_3_ and NiCl_2_ with a 3 : 1 molar ratio were dissolved in ethylene glycol. Afterward, the appropriate amount of aqueous ammonia solution (28 wt%) was added to the above solution until the pH reached 10. Then, the transparent solution was placed in an autoclave at 150 °C for 4 h. The obtained precipitate was washed twice with methanol and dried at 60 °C for 8 h. Finally, the product was calcined at 500 °C for 2 h.

### 3.3. Preparation of Co_3_O_4_/NiO@N-GQD nanocomposite

1 g citric acid and was dissolved in 20 mL deionized water, and stirred to form a clear solution. Subsequently, 0.3 mL ethylenediamine was added to the above solution and mixed to obtain a clear solution. Then, 0.1 g Co_3_O_4_/NiO nanoparticles was added to the mixture. The mixture was stirred at room temperature for 5 min. Then the solution was transferred to a 50 mL Teflon-lined stainless autoclave, which was sealed and heated to 180 °C for 12 h in an electric oven. Finally, the as-prepared nanostructured Co_3_O_4_/NiO@GQDs was obtained, which was washed several times with deionized water and ethanol, and then dried in an oven until a constant weight was achieved.

### 3.4. Preparation of Co_3_O_4_/NiO@GQD@SO_3_H nanocomposite

1 g of Co_3_O_4_/NiO@N-GQD nanocomposite was dispersed in dry CH_2_Cl_2_ (10 mL) and sonicated for 5 min. Then, chlorosulfonic acid (0.8 mL in dry CH_2_Cl_2_) was added dropwise to a cooled (ice-bath) mixture of Co_3_O_4_/NiO@N-GQDs for 30 min under N_2_ with vigorous stirring. The mixture was stirred for 120 min, while the residual HCl was removed by suction. The resulted Co_3_O_4_/NiO@GQD@SO_3_H nanocomposite was separated, washed several times with dried CH_2_Cl_2_ before drying under vacuum at 60 °C.

### 3.5. General procedure for the preparation of chromenpyrimidines

A mixture of aldehyde (1 mmol), 6-amino-1,3-dimethyluracil (1 mmol), 4-hydroxycoumarin (1 mmol) and 5 mg Co_3_O_4_/NiO@GQD@SO_3_H nanocomposite was stirred in 5 mL ethanol under reflux. The reaction was monitored by TLC. After completion of the reaction, the solution was filtered, and the heterogeneous catalyst was recovered. Water was added, and the precipitate was collected by filtration and washed with water. The crude product was recrystallized or washed with ethanol to give the pure product.

### 3.6. Spectral data

#### 6-Amino-5-((4-hydroxy-2-oxo-2H-chromen-3-yl)(phenyl) methyl)-1,3-dimethylpyrimidine-2,4(1*H*,3*H*)-dione (4a)

White solid; m.p. 220–222 °C; IR (KBr): 3432, 3234, 2964, 1695, 1664, 1616, 1569, 1444, 1356, 754 cm^−1^; ^1^H NMR (400 MHz, DMSO-*d*_6_) *δ*: 3.14 (s, 3H, –CH̲_3_), 3.40 (s, 3H, –CH̲_3_), 5.62 (s, 1H, –CH̲), 7.16–7.85 (m, 11H, ArH, –NH̲_2_), 13.99 (s, 1H, –OH̲); ^13^C NMR (100 MHz, DMSO-*d*_6_) *δ*: 28.68, 31.02, 36.53, 87.26, 105.11, 116.62, 117.41, 124.17, 124.76, 126.14, 126.80, 128.55, 132.87, 138.74, 150.50, 152.44, 155.63, 164.27, 164.55, 166.27; anal. calcd for C_22_H_19_N_3_O_5_: C, 65.18%; H, 4.72%; N, 10.37%. Found: C, 65.20%; H, 4.59%; N, 10.25%.

#### 6-Amino-5-((4-hydroxy-2-oxo-2H-chromen-3-yl)(4-chlorophenyl) methyl)-1,3-dimethylpyrimidine-2,4(1*H*,3*H*)-dione (4b)

White solid; m.p. 218–220 °C; IR (KBr): 3405, 3211, 2921, 1695, 1663, 1617, 1493, 1448, 763 cm^−1^; ^1^H NMR (400 MHz, DMSO-*d*_6_) *δ*: 3.14 (s, 3H), 3.37 (s, 3H), 5.61 (s, 1H), 7.17–7.85 (m, 10H, ArH and NH_2_), 13.98 (s, 1H, OH); ^13^C NMR (100 MHz, DMSO-*d*_6_) *δ*: 28.72, 31.14, 36.17, 86.98, 104.96, 116.60, 117.35, 124.20, 124.82, 128.43, 128.96, 130.78, 132.96, 138.02, 150.52, 152.40, 155.62, 164.25, 164.52, 166.14; anal. calcd for C_22_H_18_ClN_3_O_5_: C, 60.07%; H, 4.12%; N, 9.55%. Found: C, 60.15%; H, 4.18%; N, 9.42%.

#### 6-Amino-5-((4-hydroxy-2-oxo-2H-chromen-3-yl)(4-methoxy phenyl) methyl)-1,3-dimethylpyrimidine-2,4(1*H*,3*H*)-dione (4c)

Light yellow solid, m.p.: 178–180 °C; IR (KBr): 3413, 3238, 2952, 1695, 1613, 1568, 1508, 1450, 1253, 767 cm^−1^, ^1^H NMR (400 MHz, DMSO-*d*_6_) *δ*: 3.14 (s, 3H), 3.37 (s, 3H), 3.69 (s, 3H), 5.61 (s, 1H), 6.78–6.80 (d, *J* = 8.0 Hz, 2H), 7.03–7.05 (d, *J* = 8.0 Hz, 2H), 7.32–7.84 (m, 6H), 13.98 (s, 1H, OH); ^13^C NMR (100 MHz, DMSO-*d*_6_) *δ*: 28.66, 31.02, 35.83, 55.43, 87.45, 105.43, 113.95, 116.62, 117.42, 124.15, 124.75, 127.90, 130.32, 132.82, 150.56, 152.42, 155.57, 157.88, 164.20, 164.54, 166.29; anal. calcd for C_23_H_21_N_3_O_6_: C, 63.44%; H, 4.86%; N, 9.65%. Found: C, 63.49%; H, 4.74%; N, 9.59%.

#### 6-Amino-5-((4-hydroxy-2-oxo-2H-chromen-3-yl)(4-nitrophenyl) methyl)-1,3-dimethylpyrimidine-2,4(1*H*,3*H*)-dione (4d)

Light yellow solid; m.p.: 240–242 °C; IR (KBr): 3396, 3210, 2903, 1685, 1619, 1571, 1514, 1346, 769 cm^−1^; ^1^H NMR (400 MHz, DMSO-*d*_6_) *δ*: 3.14 (s, 3H), 3.49 (s, 3H), 5.75 (s, 1H), 7.35–8.10 (m, 10H), 13.96 (s, 1H, OH); ^13^C NMR (100 MHz, DMSO-*d*_6_) *δ*: 28.69, 31.05, 36.52, 86.48, 104.72, 117.30, 121.45, 121.63, 124.22, 130.00, 133.04, 134.12, 141.74, 148.41, 150.46, 152.47, 155.80, 164.34, 164.55, 165.98; anal. calcd for C_22_H_18_N_4_O_7_: C, 58.67%; H, 4.03%; N, 12.44%; found C, 58.72%; H, 4.09%; N, 12.52%.

#### 6-Amino-5-((4-hydroxy-2-oxo-2H-chromen-3-yl)(3-nitrophenyl) methyl)-1,3-dimethylpyrimidine-2,4(1*H*,3*H*)-dione (4e)

Light yellow solid; m.p.: 230–232 °C; IR (KBr): 3460, 3209, 2923, 1699, 1673, 1523, 1437, 1343, 772 cm^−1^; ^1^H NMR (400 MHz, DMSO-*d*_6_) *δ*: 3.14 (s, 3H), 3.38 (s, 3H), 5.76 (s, 1H), 7.36–8.06 (m, 10H), 13.97 (s, 1H, OH); ^13^C NMR (100 MHz, DMSO-*d*_6_) *δ*: 28.69, 31.05, 36.52, 86.48, 104.70, 116.54, 117.35, 121.42, 121.62, 124.80, 124.24, 130.14, 133.24, 134.22, 141.75, 148.45, 150.49, 152.42, 155.92, 164.42, 164.45, 165.85; anal. calcd for C_22_H_18_N_4_O_7_: C, 58.67%; H, 4.03%; N, 12.44%; found C, 58.75%; H, 4.12%; N, 12.54%.

#### 6-Amino-5-((4-hydroxy-2-oxo-2H-chromen-3-yl)(4-hydroxyphenyl) methyl)-1,3-dimethylpyrimidine-2,4(1*H*,3*H*)-dione (4f)

White solid; m.p. 230–232 °C; IR (KBr): 3364, 3219, 1695, 1671, 1569, 1509, 1444, 1358, 769 cm^−1^; ^1^H NMR (400 MHz, DMSO-*d*_6_) *δ*: 3.14 (s, 3H), 3.49 (s, 3H), 5.51 (s, 1H), 6.60–6.62 (d, *J* = 8.0 Hz, 2H), 6.90–6.92 (d, *J* = 8.0 Hz, 2H), 7.29–7.84 (m, 6H), 9.14 (s, 1H, OH), 13.97 (s, 1H, OH); ^13^C NMR (100 MHz, DMSO-*d*_6_) *δ*: 28.64, 30.96, 35.74, 87.54, 105.45, 115.35, 116.55, 117.40, 124.12, 124.73, 127.77, 128.41, 132.79, 150.50, 152.35, 155.43, 155.76, 164.17, 164.49, 166.27. Anal. calcd for C_22_H_19_N_3_O_6_: C, 62.70%; H, 4.54%; N, 9.97%; found C, 62.78%; H, 4.59%; N, 10.05%.

#### 6-Amino-5-((4-hydroxy-2-oxo-2H-chromen-3-yl)(3-hydroxyphenyl) methyl)-1,3-dimethylpyrimidine-2,4(1*H*,3*H*)-dione (4g)

White solid; m.p. 232–234 °C; IR (KBr): 3429, 3306, 2957, 1695, 1670, 1617, 1569, 1447, 1354, 760 cm^−1^; ^1^H NMR (400 MHz, DMSO-*d*_6_) *δ*: 3.15 (s, 3H), 3.38 (s, 3H), 5.55 (s, 1H), 6.53–7.67 (m, 10H), 9.12 (s, 1H, OH), 14.03 (s, 1H, OH); ^13^C NMR (100 MHz, DMSO-*d*_6_) *δ*: 28.72, 30.94, 35.72, 87.55, 104.55, 115.34, 116.52, 116.58, 117.42, 124.14, 124.25, 124.78, 127.75, 128.43, 132.79, 151.22, 152.34, 155.42, 155.78, 164.15, 164.45, 166.28. Anal. calcd for C_22_H_19_N_3_O_6_: C, 62.70%; H, 4.54%; N, 9.97%; found C, 62.72%; H, 4.55%; N, 10.09%.

#### 6-Amino-5-((4-hydroxy-2-oxo-2H-chromen-3-yl)(2-hydroxyphenyl) methyl)-1,3-dimethylpyrimidine-2,4(1*H*,3*H*)-dione (4h)

White solid; m.p. 240–242 °C; IR (KBr): 3432, 3306, 2958, 1695, 1607, 1617, 1447, 760 cm^−1^; ^1^H NMR (400 MHz, DMSO-*d*_6_) *δ*: 3.15 (s, 3H), 3.38 (s, 3H), 5.55 (s, 1H), 6.63–7.87 (m, 10H), 9.11 (s, 1H, OH), 14.03 (s, 1H, OH); ^13^C NMR (100 MHz, DMSO-*d*_6_) *δ*: 28.67, 31.02, 36.40, 87.35, 104.98, 113.16, 113.69, 116.58, 116.59, 117.48, 124.23, 124.81, 124.83, 129.47, 132.93, 140.27, 150.51, 152.36, 155.52, 157.72, 164.48, 166.23. Anal. calcd for C_22_H_19_N_3_O_6_: C, 62.70%; H, 4.54%; N, 9.97%; found C, 62.82%; H, 4.62%; N, 9.98%.

#### 6-Amino-5-((4-hydroxy-2-oxo-2H-chromen-3-yl)(4-methylphenyl) methyl)-1,3-dimethylpyrimidine-2,4(1*H*,3*H*)-dione (4i)

White solid; m.p. 202–204 °C; IR (KBr): 3411, 3212, 2922, 1697, 1665, 1618, 1569, 1353, 764 cm^−1^; ^1^H NMR (400 MHz, DMSO-*d*_6_) *δ*: 2.24 (s, 3H), 3.14 (s, 3H), 3.37 (s, 3H), 5.57 (s, 1H), 6.96–7.85 (m, 10H), 13.98 (s, 1H, OH); ^13^C NMR (100 MHz, DMSO-*d*_6_) *δ*: 20.60, 28.32, 30.64, 35.74, 87.30, 104.72, 116.25, 123.72, 124.17, 124.42, 126.37, 128.82, 132.58, 134.74, 135.13, 150.18, 152.02, 155.12, 164.15, 165.93, 167.75; anal. calcd for C_23_H_21_N_3_O_5_: C, 65.86%; H, 5.05%; N, 10.02%; found C, 65.92%; H, 5.15%; N, 10.08%.

#### 6-Amino-5-((4-hydroxy-2-oxo-2H-chromen-3-yl)(3-methylphenyl) methyl)-1,3-dimethylpyrimidine-2,4(1*H*,3*H*)-dione (4j)

White solid; m.p. 200–202 °C; IR (KBr): 3403, 3229, 2923, 1699, 1695, 1445, 758 cm^−1^; ^1^H NMR (400 MHz, DMSO-*d*_6_) *δ*: 2.21 (s, 3H), 3.15 (s, 3H), 3.38 (s, 3H), 5.59 (s, 1H), 6.92–7.86 (m, 10H), 13.97 (s, 1H, OH); ^13^C NMR (100 MHz, DMSO-*d*_6_) *δ*: 21.63, 28.66, 30.98, 36.37, 87.34, 105.11, 116.58, 117.37, 123.91, 124.18, 124.77, 126.87, 127.29, 128.42, 132.85, 137.50, 138.66, 150.50, 152.37, 155.52, 164.26, 164.54, 166.25; anal. calcd for C_23_H_21_N_3_O_5_: C, 65.86%; H, 5.05%; N, 10.02%; found C, 65.96%; H, 5.18%; N, 10.12%.

#### 6-Amino-5-((4-hydroxy-2-oxo-2H-chromen-3-yl)(4-bromophenyl) methyl)-1,3-dimethylpyrimidine-2,4(1*H*,3*H*)-dione (4k)

White solid; m.p. 236–238 °C; IR (KBr): 3404, 3212, 2921, 1675, 1663, 1616, 1488, 1446, 761 cm^−1^; ^1^H NMR (400 MHz, DMSO-*d*_6_) *δ*: 3.13 (s, 3H), 3.37 (s, 3H), 5.58 (s, 1H), 7.11–7.84 (m, 10H), 13.97 (s, 1H, OH); ^13^C NMR (100 MHz, DMSO-*d*_6_) *δ*: 28.75, 31.17, 36.19, 86.95, 104.93, 116.63, 117.34, 124.22, 124.85, 128.40, 128.93, 130.79, 132.94, 138.08, 150.55, 152.42, 155.67, 164.28, 164.54, 166.16; anal. calcd for C_22_H_18_BrN_3_O_5_: C, 54.56%; H, 3.75%; N, 8.68%. Found: C, 54.62%; H, 3.83%; N, 8.75%.

#### 6-Amino-5-((4-hydroxy-2-oxo-2H-chromen-3-yl)(4-isopropylphenyl) methyl)-1,3-dimethylpyrimidine-2,4(1*H*,3*H*)-dione (4l)

White solid; m.p. 257–259 °C; IR (KBr): 3463, 3230, 2958, 1695, 1671, 1616, 1569, 1446, 1343, 762 cm^−1^; ^1^H NMR (400 MHz, DMSO-*d*_6_) *δ*: 1.15 (s, 6H), 2.77 (m, 1H), 3.15 (s, 3H), 3.38 (s, 3H), 5.57 (s, 1H), 7.02–7.85 (m, 10H), 13.99 (s, 1H, OH); ^13^C NMR (100 MHz, DMSO-*d*_6_) *δ*: 23.8, 28.1, 30.3, 32.9, 35.6, 87.1, 104.4, 115.8, 116.9, 123.7, 123.9, 125.8, 126.1, 131.9, 135.2, 145.5, 149.9, 151.9, 155.0, 163.8, 164.1, 168.3; anal. calcd for C_25_H_25_N_3_O_5_: C, 67.10%; H, 5.63%; N, 9.39%; found C, 67.19%; H, 5.75%; N, 9.45%.

#### 6-Amino-5-((4-hydroxy-2-oxo-2H-chromen-3-yl)(2-bromophenyl) methyl)-1,3-dimethylpyrimidine-2,4(1*H*,3*H*)-dione (4m)

White solid; m.p. 210–212 °C; IR (KBr): 3402, 3210, 2925, 1676, 1665, 1618, 1489, 1447, 765 cm^−1^; ^1^H NMR (400 MHz, DMSO-*d*_6_) *δ*: 3.12 (s, 3H), 3.35 (s, 3H), 5.57 (s, 1H), 7.12–7.86 (m, 10H), 13.96 (s, 1H, OH); ^13^C NMR (100 MHz, DMSO-*d*_6_) *δ*: 28.74, 31.16, 36.10, 86.94, 104.92, 116.63, 117.29, 124.25, 124.82, 126.35, 127.52, 128.40, 128.92, 130.75, 132.96, 138.05, 150.56, 152.47, 155.65, 164.22, 164.55, 166.14; anal. calcd for C_22_H_18_BrN_3_O_5_: C, 54.56%; H, 3.75%; N, 8.68%. Found: C, 54.65%; H, 3.86%; N, 8.77%.

#### 6-Amino-5-((4-hydroxy-2-oxo-2H-chromen-3-yl)(3-bromophenyl) methyl)-1,3-dimethylpyrimidine-2,4(1*H*,3*H*)-dione (4n)

White solid; m.p. 245–247 °C; IR (KBr): 3405, 3210, 2924, 1673, 1665, 1617, 1489, 1445, 764 cm^−1^; ^1^H NMR (400 MHz, DMSO-*d*_6_) *δ*: 3.13 (s, 3H), 3.35 (s, 3H), 5.59 (s, 1H), 7.10–7.89 (m, 10H), 13.98 (s, 1H, OH); ^13^C NMR (100 MHz, DMSO-*d*_6_) *δ*: 28.74, 31.18, 36.17, 86.95, 104.94, 116.62, 117.31, 124.26, 124.85, 125.33, 126.54, 128.42, 128.94, 130.72, 132.90, 138.07, 150.52, 152.43, 155.64, 164.22, 164.50, 166.12; anal. calcd for C_22_H_18_BrN_3_O_5_: C, 54.56%; H, 3.75%; N, 8.68%. Found: C, 54.65%; H, 3.87%; N, 8.79%.

## 4. Conclusions

In conclusion, we reported an efficient method for the synthesis of chromenpyrimidines using the Co_3_O_4_/NiO@GQD@SO_3_H nanocomposite as a superior catalyst under reflux conditions. The new catalyst was characterized *via* FT-IR, SEM, XRD, EDS, TGA, BET and VSM. The current method provides obvious advantages, including environmental friendliness, short reaction time, reusability of the catalyst, low catalyst loading and simple workup procedure.

## Conflicts of interest

There are no conflicts to declare.

## Supplementary Material

RA-009-C9RA05896F-s001
