# Supplementary material for: Co3O4/NiO@GQD@SO3H nanocomposite as a superior catalyst for the synthesis of chromenpyrimidines
Source: RSC Adv. 2019 Nov 15;9(64):37344–54. doi: 10.1039/c9ra05896f (PMC9075579; doi:10.1039/c9ra05896f)

Electronic Supplementary Material (ESI) for RSC Advances.
This journal is © The Royal Society of Chemistry 2019

Supplementary information

Co_3O_4/NiO@GQDs@SO_3H nanocomposite as a superior catalyst for the synthesis of chromenpyrimidines

Javad Safaei-Ghomi*, Zahra Omidshafiei
Department of Organic Chemistry, Faculty of Chemistry, University of Kashan, Kashan, P.O. Box 87317-51167, I. R.Iran Corresponding author. E-mail addresses: safaei@kashanu.ac.ir, Fax: +98-31-55912397; Tel.: +98-31-55912385

General procedure for the preparation of chromenpyrimidines:

A mixture of aldehyde (1 mmol), 6-amino-1,3-dimethyluracil (1 mmol), 4-hydroxy coumarine (1 mmol) and 5 mg Co_3O_4/NiO@GQDs@SO_3H nanocomposite were stirred in 5 mL ethanol under reflux condition. The reaction was monitored by TLC. After completion of the reaction, the solution was filtered and the heterogeneous catalyst was recovered. Water was added, and the precipitate was collected by filtration and washed with water. The crude product was recrystallized or washed with ethanol to give the pure product.

The conversion is described as ratios of how much of a reactant has reacted.

Conversion efficiency = $\frac{The\ amount\ of\ \ Limiting\ reagent\ consumed}{The\ amount\ of\ Limiting\ reagent\ in} \times 100$

FT-IR: 6-Amino-5-((4-hydroxy-2-oxo-2H-chromen-3-yl) (phenyl) methyl)-1,3-dimethylpyrimidine-2,4(1H,3H)-dione (4a):

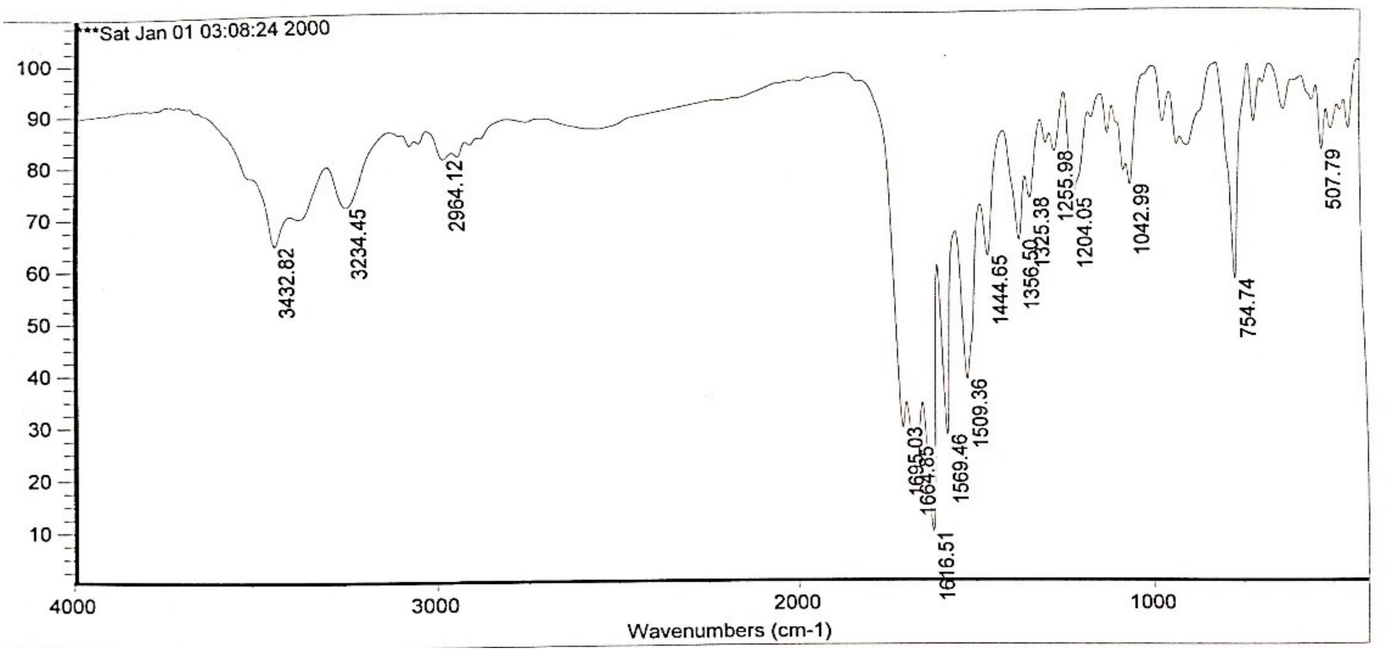

H NMR: 6-Amino-5-((4-hydroxy-2-oxo-2H-chromen-3-yl) (phenyl) methyl)-1,3-dimethylpyrimidine-2,4(1H,3H)-dione (4a):

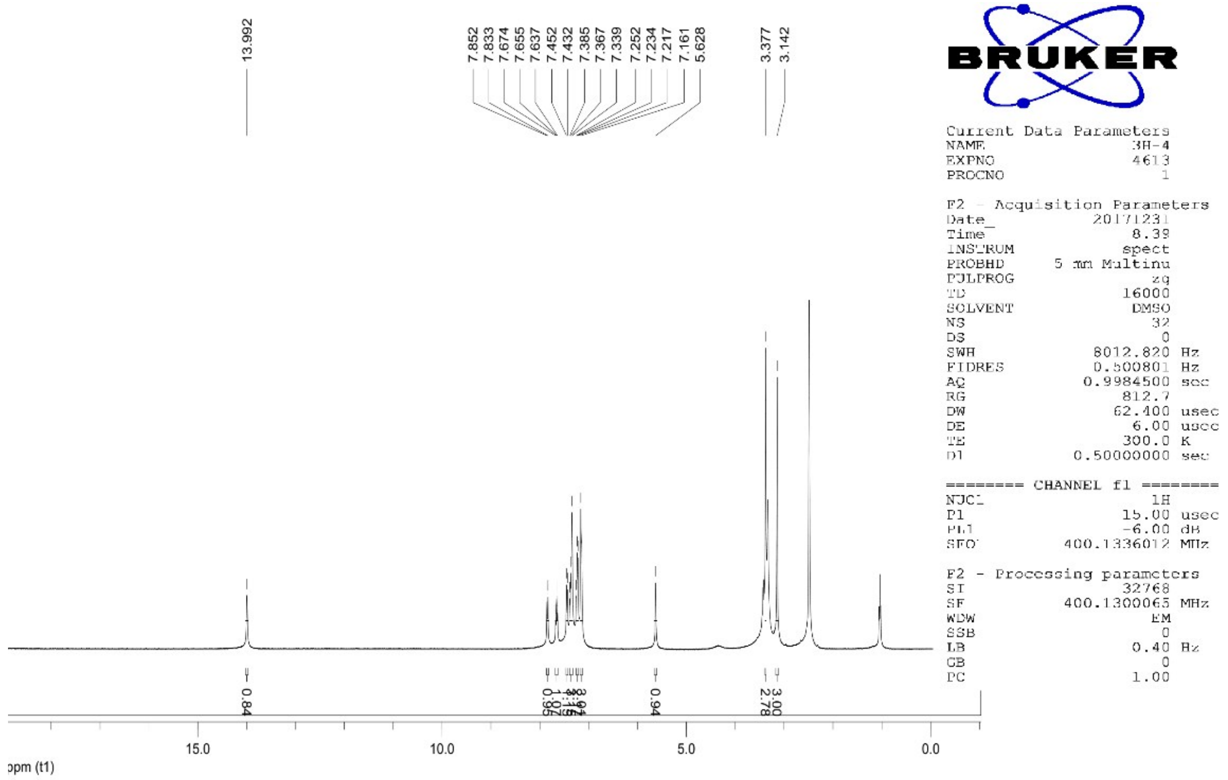

FT-IR: 6-Amino-5-((4-hydroxy-2-oxo-2H-chromen-3-yl) (4-chlorophenyl) methyl)-1,3-dimethylpyrimidine-2,4(1H,3H)-dione (4b):

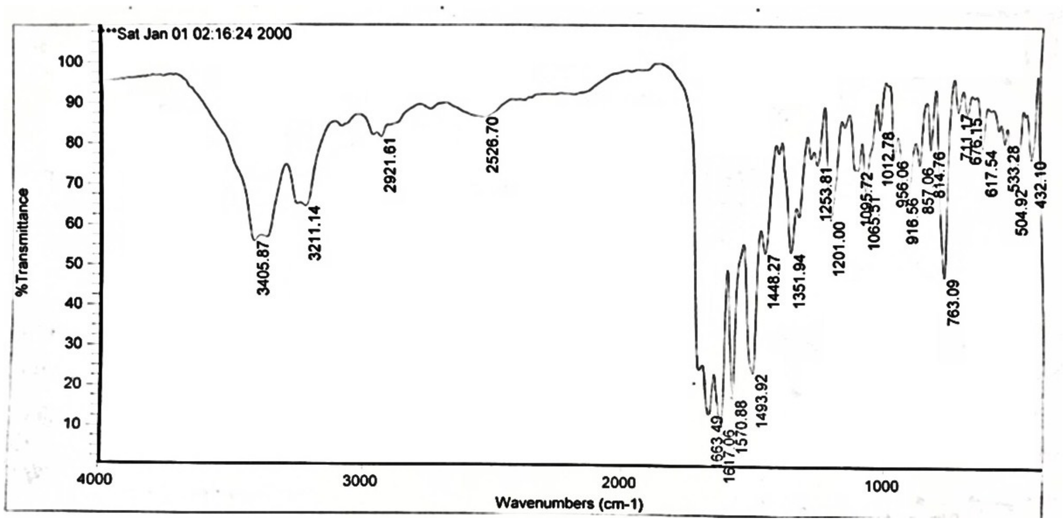

H NMR: 6-Amino-5-((4-hydroxy-2-oxo-2H-chromen-3-yl) (4-chlorophenyl) methyl)-1,3-dimethylpyrimidine-2,4(1H,3H)-dione (4b):

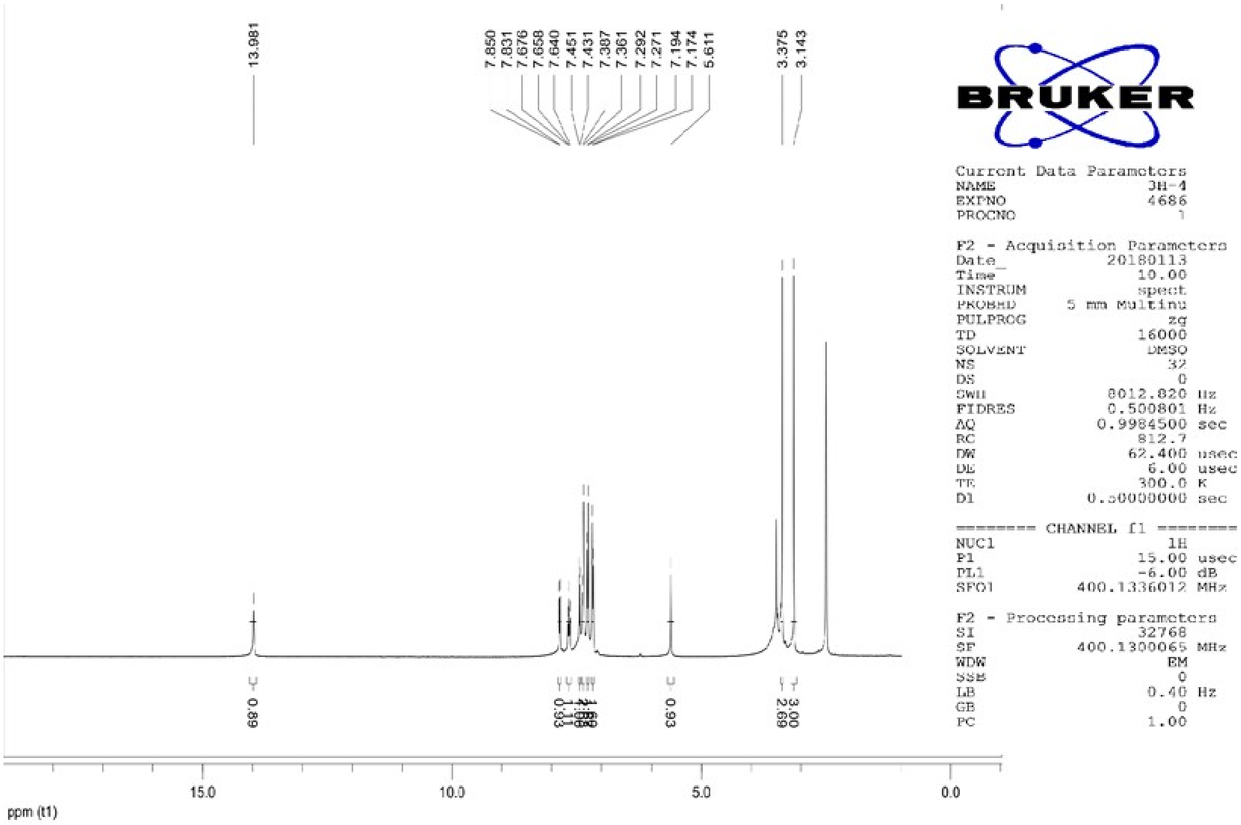

FT-IR: 6-Amino-5-((4-hydroxy-2-oxo-2H-chromen-3-yl) (4-methoxyphenyl) methyl)-1,3-dimethylpyrimidine-2,4(1H,3H)-dione (4c):

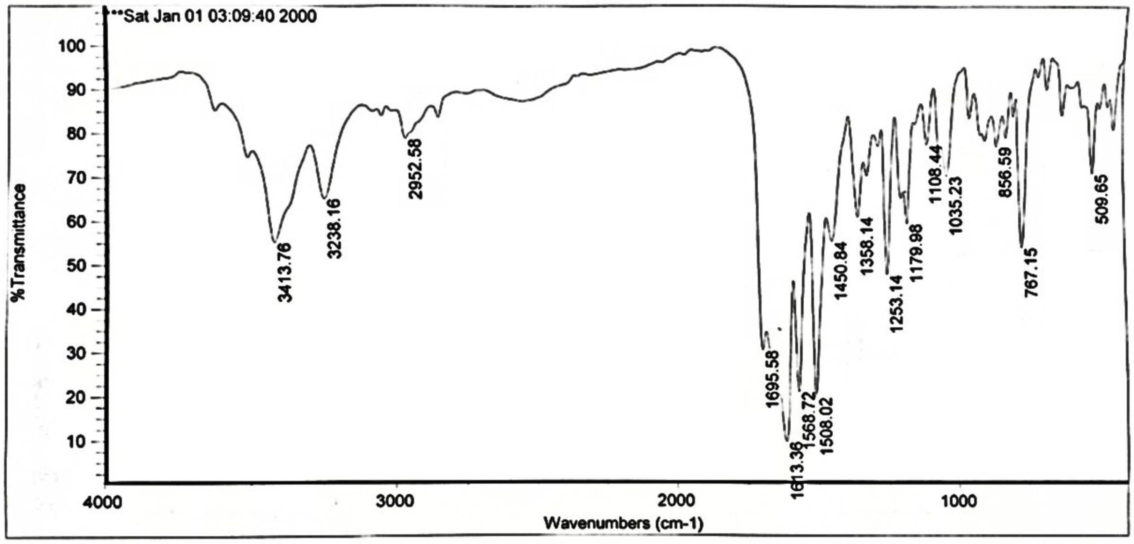

H NMR: 6-Amino-5-((4-hydroxy-2-oxo-2H-chromen-3-yl) (4-methoxyphenyl) methyl)-1,3-dimethylpyrimidine-2,4(1H,3H)-dione (4c):

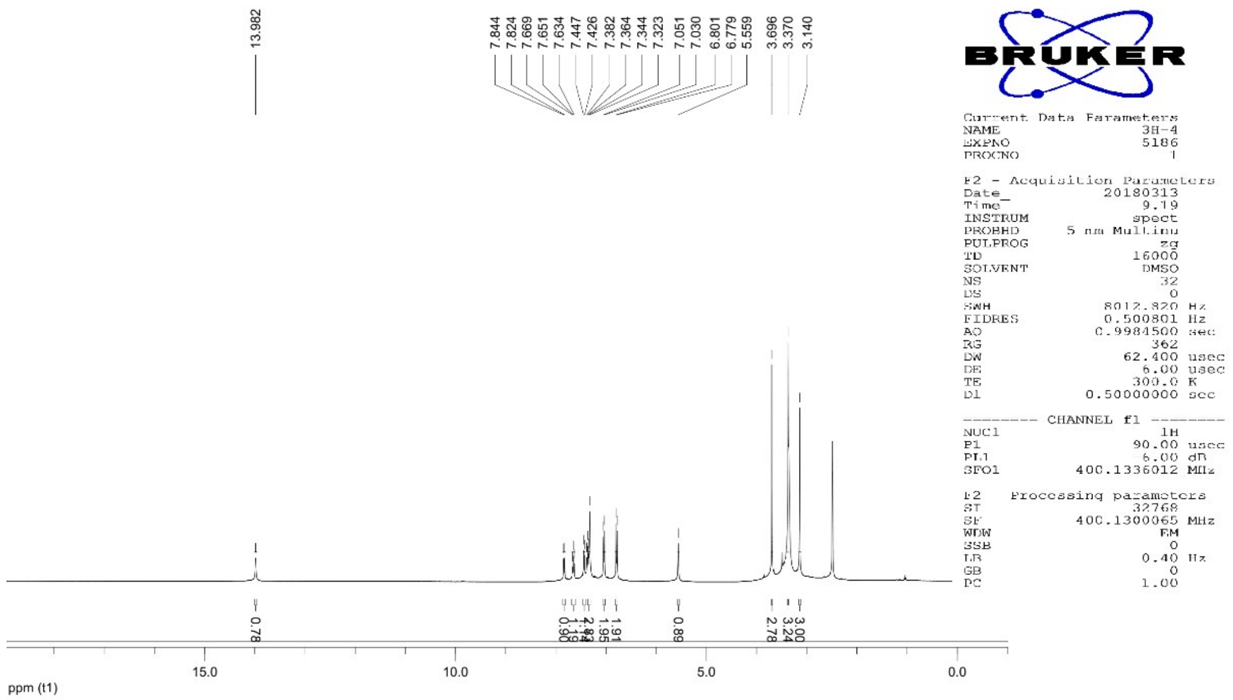

FT-IR: 6-Amino-5-((4-hydroxy-2-oxo-2H-chromen-3-yl) (4-nitrophenyl) methyl)-1,3-dimethylpyrimidine-2,4(1H,3H)-dione (4d):

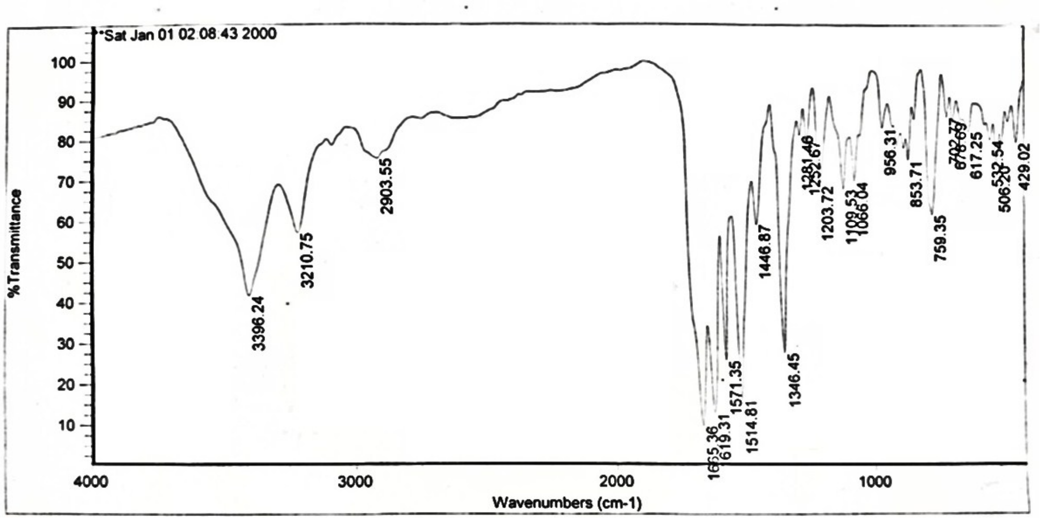

H NMR: 6-Amino-5-((4-hydroxy-2-oxo-2H-chromen-3-yl) (4-nitrophenyl) methyl)-1,3-dimethylpyrimidine-2,4(1H,3H)-dione (4d):

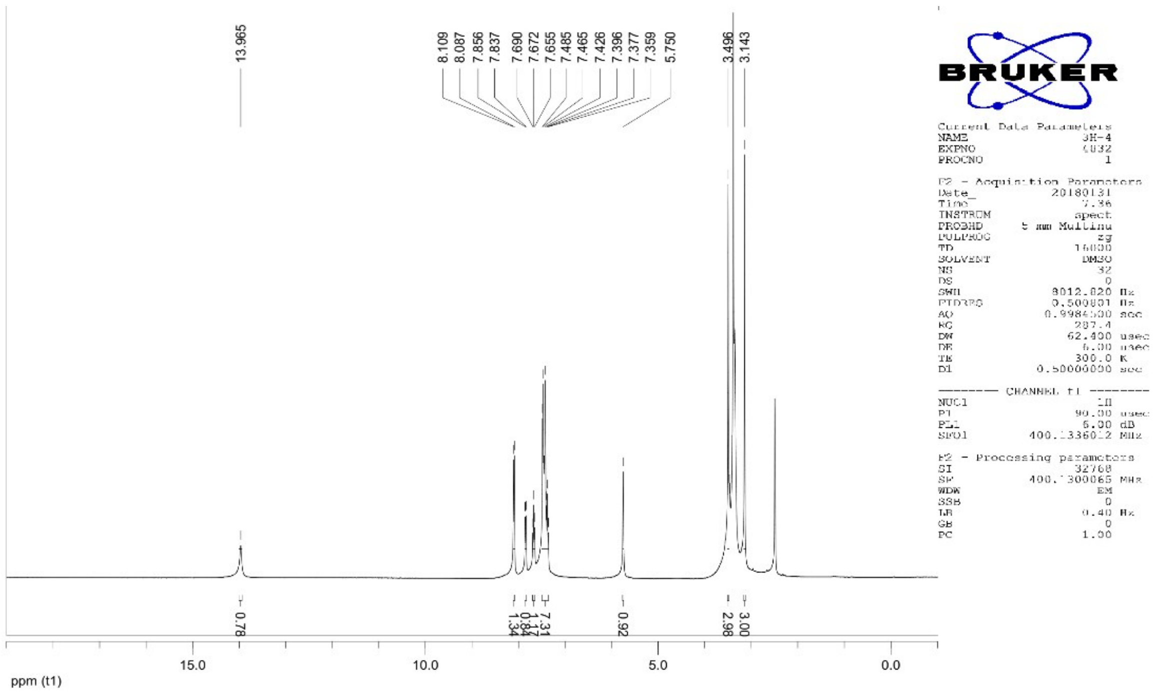

^{13}C NMR: 6-Amino-5-((4-hydroxy-2-oxo-2H-chromen-3-yl) (4-nitrophenyl) methyl)-1,3-dimethylpyrimidine-2,4(1H,3H)-dione (4d):

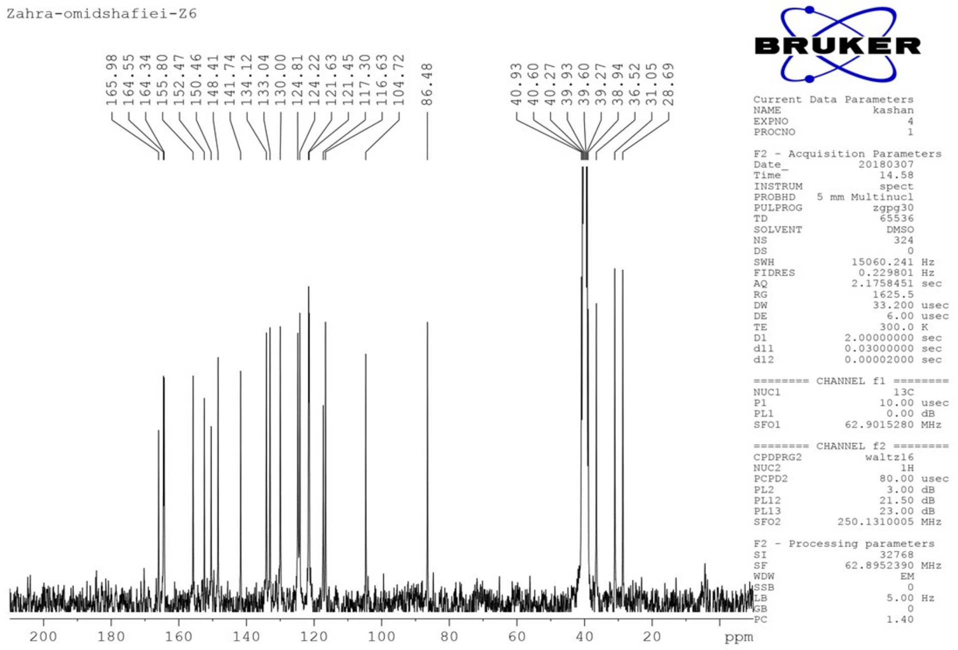

FT-IR: 6-Amino-5-((4-hydroxy-2-oxo-2H-chromen-3-yl) (3-nitrophenyl) methyl)-1,3-dimethylpyrimidine-2,4(1H,3H)-dione (4e):

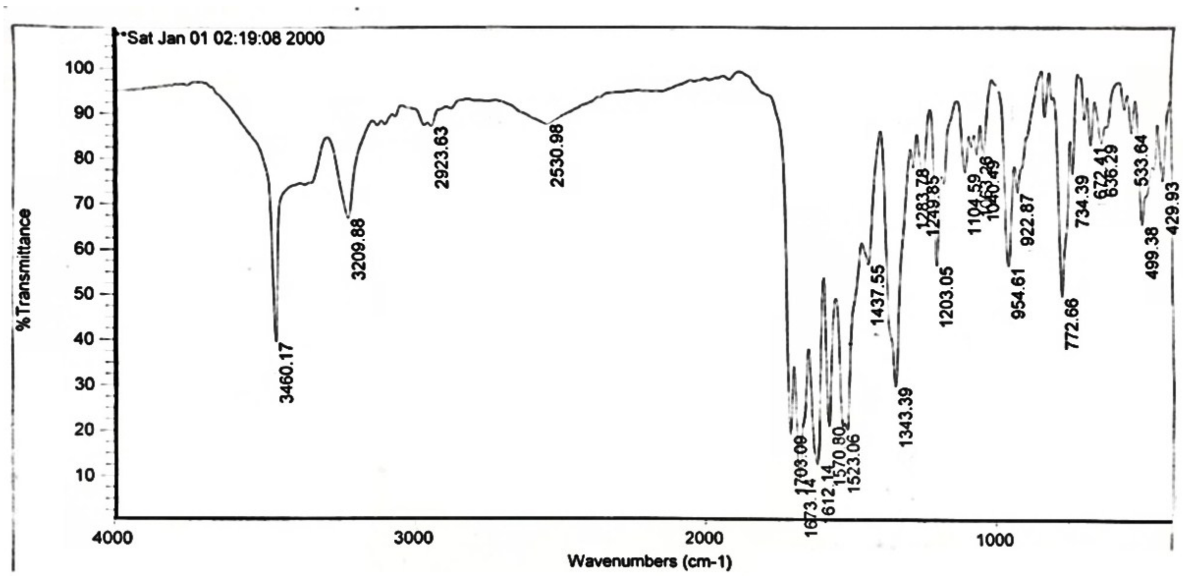

H NMR: 6-Amino-5-((4-hydroxy-2-oxo-2H-chromen-3-yl) (3-nitrophenyl) methyl)-1,3-dimethylpyrimidine-2,4(1H,3H)-dione (4e):

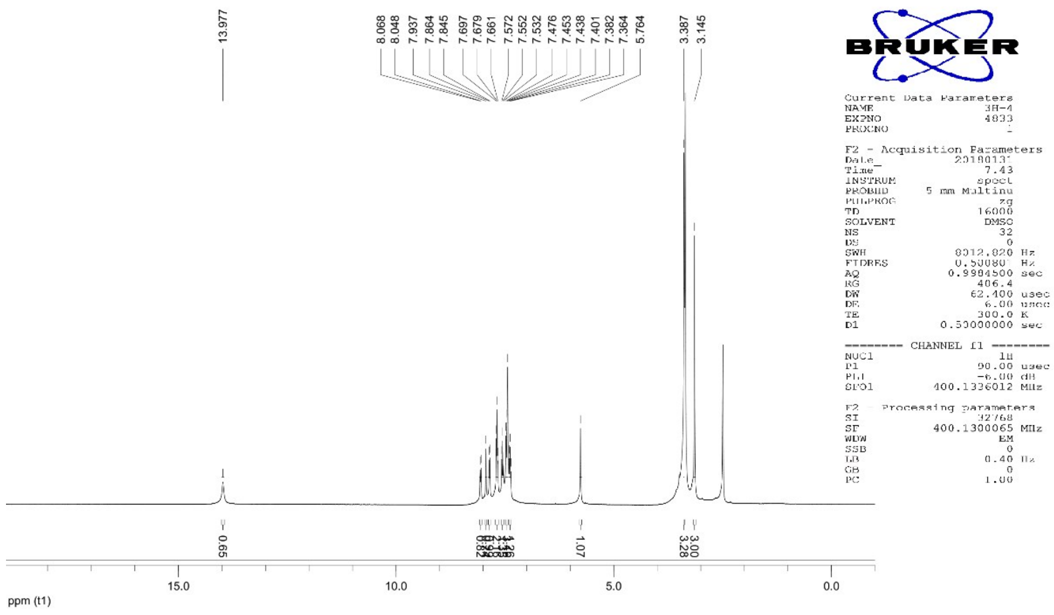

FT-IR: 6-Amino-5-((4-hydroxy-2-oxo-2H-chromen-3-yl) (4-hydroxyphenyl) methyl)-1,3-dimethylpyrimidine-2,4(1H,3H)-dione (4f):

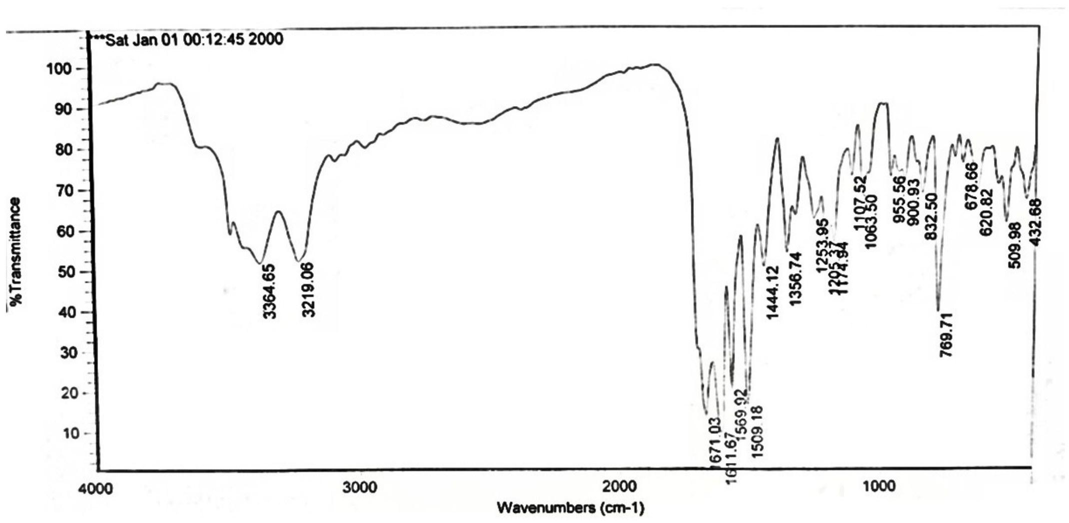

H NMR: 6-Amino-5-((4-hydroxy-2-oxo-2H-chromen-3-yl) (4-hydroxyphenyl) methyl)-1,3-dimethylpyrimidine-2,4(1H,3H)-dione (4f):

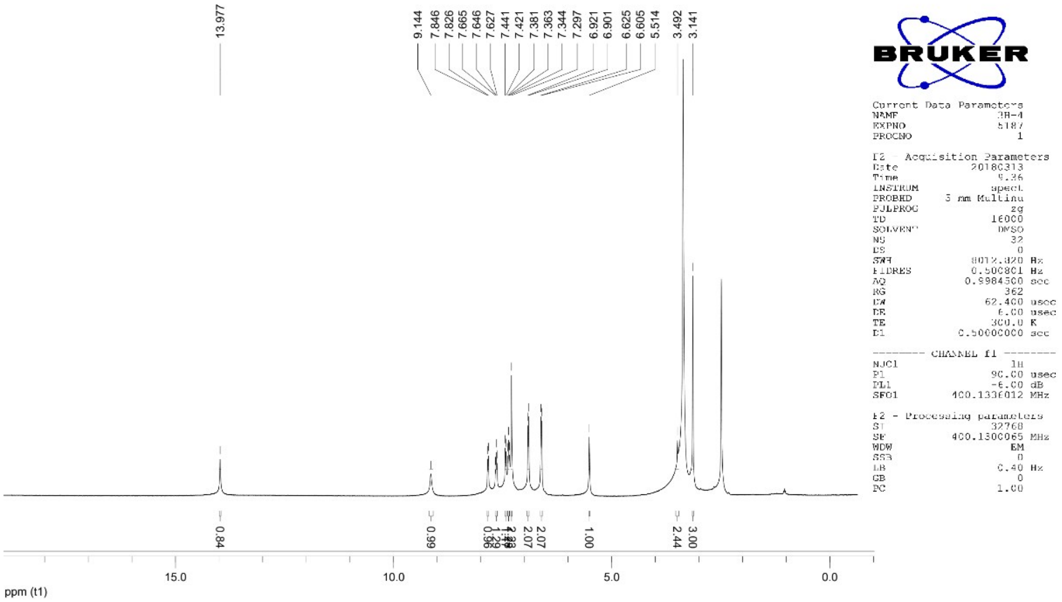

^{13}C NMR: 6-Amino-5-((4-hydroxy-2-oxo-2H-chromen-3-yl) (4-hydroxyphenyl) methyl)-1,3-dimethylpyrimidine-2,4(1H,3H)-dione (4f):

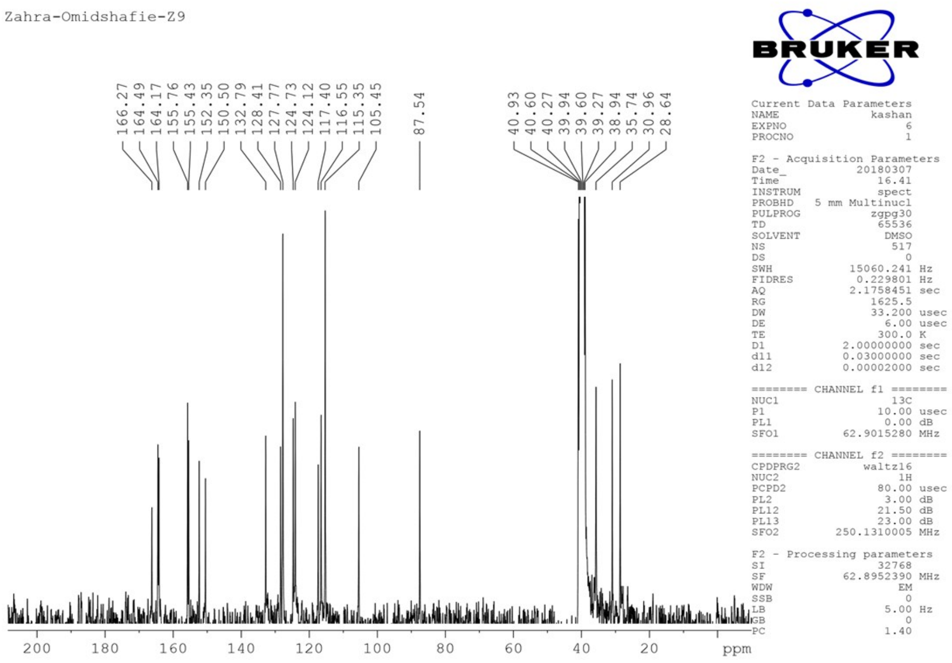

FT-IR: 6-Amino-5-((4-hydroxy-2-oxo-2H-chromen-3-yl) (3-hydroxyphenyl) methyl)-1,3-dimethylpyrimidine-2,4(1H,3H)-dione (4g):

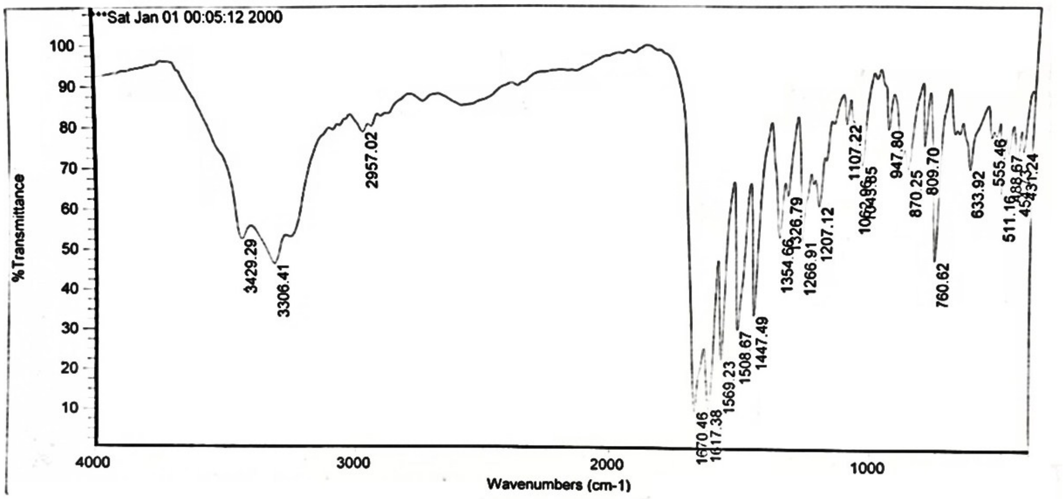

H NMR: 6-Amino-5-((4-hydroxy-2-oxo-2H-chromen-3-yl) (3-hydroxyphenyl) methyl)-1,3-dimethylpyrimidine-2,4(1H,3H)-dione (4g):

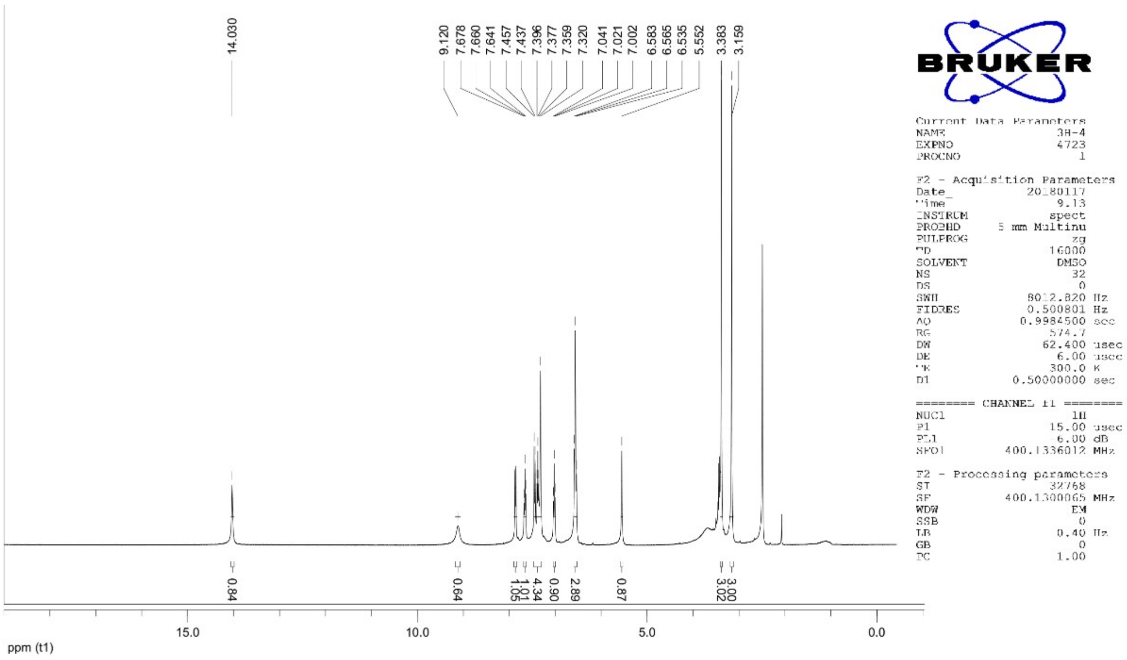

FT-IR: 6-Amino-5-((4-hydroxy-2-oxo-2H-chromen-3-yl) (2-hydroxyphenyl) methyl)-1,3-dimethylpyrimidine-2,4(1H,3H)-dione (4h):

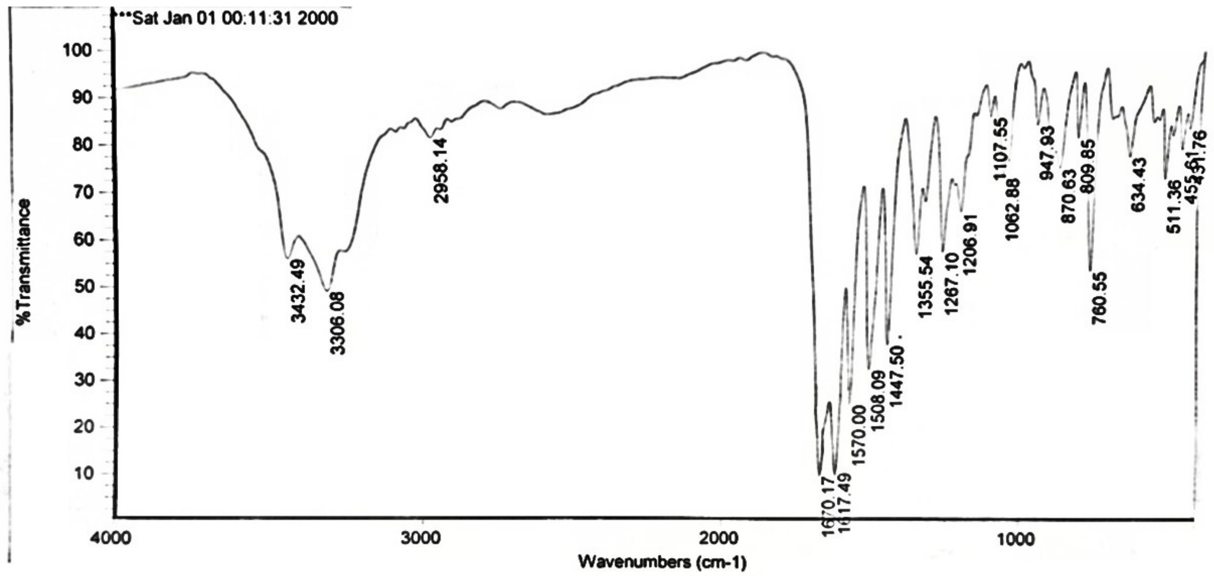

H NMR: 6-Amino-5-((4-hydroxy-2-oxo-2H-chromen-3-yl) (2-hydroxyphenyl) methyl)-1,3-dimethylpyrimidine-2,4(1H,3H)-dione (4h):

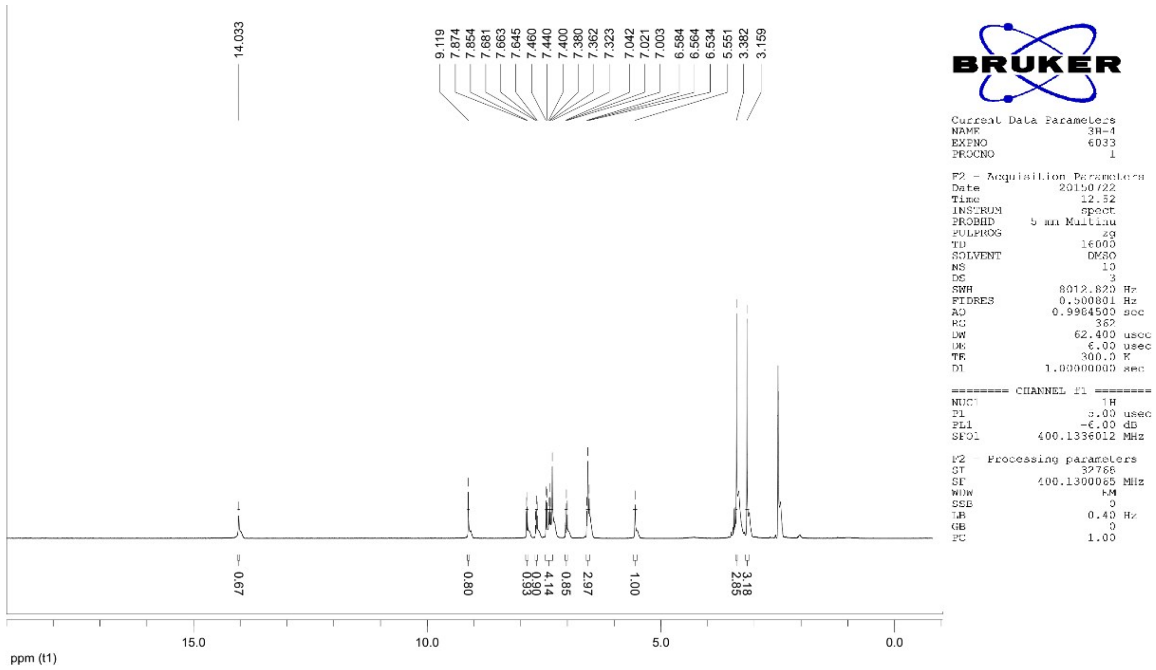

C NMR: 6-Amino-5-((4-hydroxy-2-oxo-2H-chromen-3-yl) (2-hydroxyphenyl) methyl)-1,3-dimethylpyrimidine-2,4(1H,3H)-dione (4h):

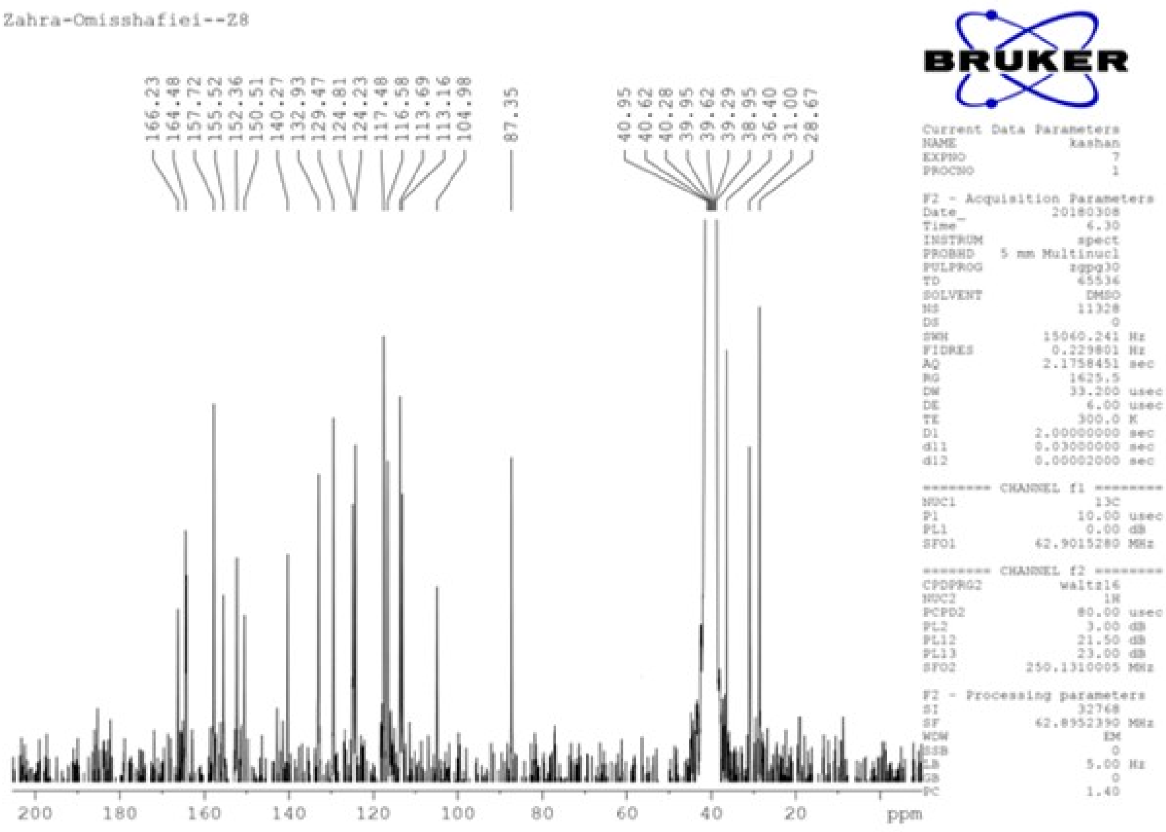

FT-IR: 6-Amino-5-((4-hydroxy-2-oxo-2H-chromen-3-yl) (4-methylphenyl) methyl)-1,3-dimethylpyrimidine-2,4(1H,3H)-dione (4i):

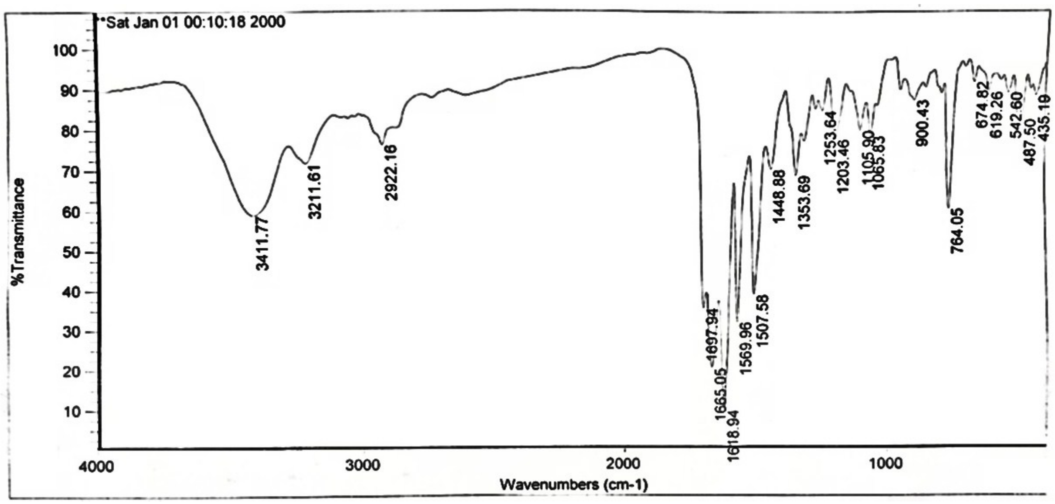

H NMR: 6-Amino-5-((4-hydroxy-2-oxo-2H-chromen-3-yl) (4-methylphenyl) methyl)-1,3-dimethylpyrimidine-2,4(1H,3H)-dione (4i):

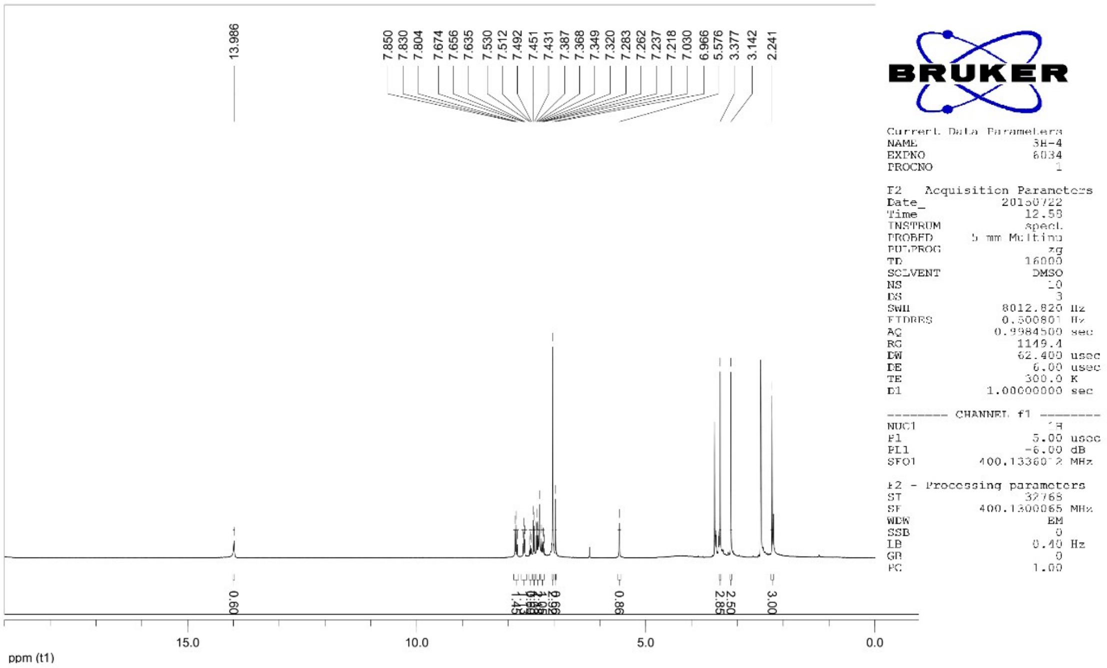

FT-IR: 6-Amino-5-((4-hydroxy-2-oxo-2H-chromen-3-yl) (3-methylphenyl) methyl)-1,3-dimethylpyrimidine-2,4(1H,3H)-dione (4j):

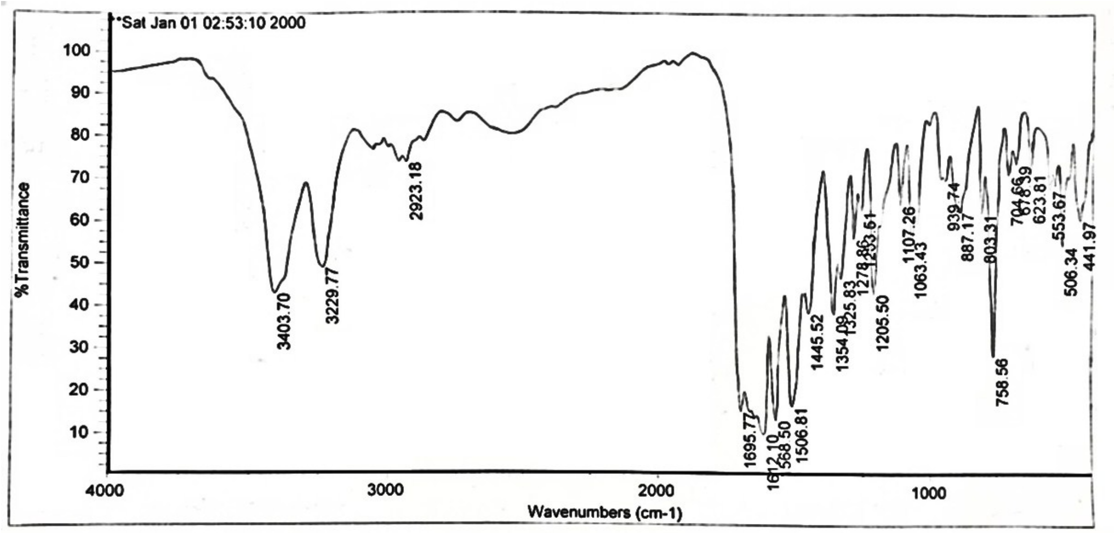

H NMR: 6-Amino-5-((4-hydroxy-2-oxo-2H-chromen-3-yl) (3-methylphenyl) methyl)-1,3-dimethylpyrimidine-2,4(1H,3H)-dione (4j):

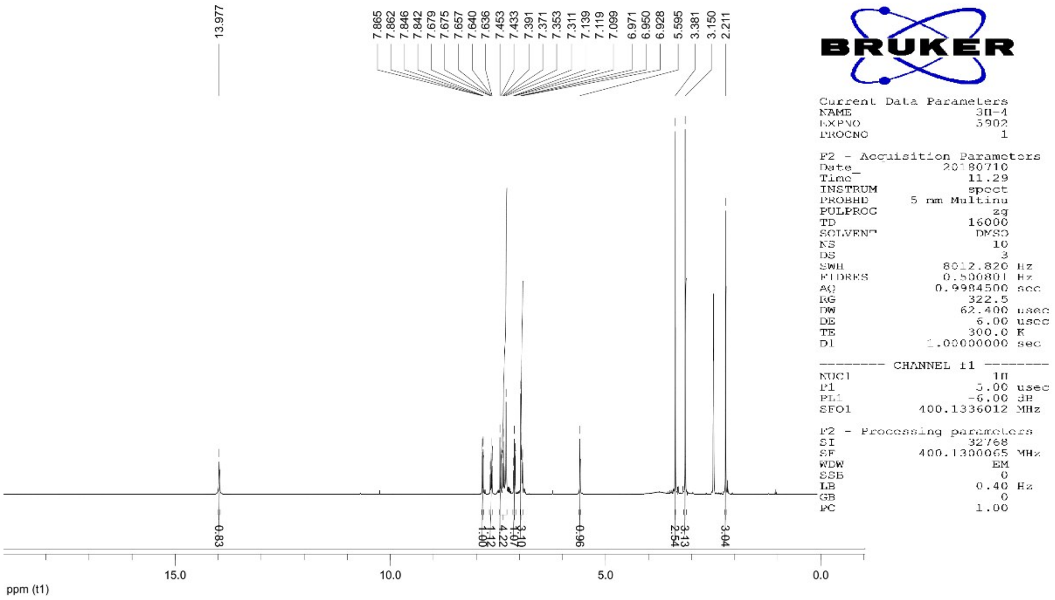

C NMR: 6-Amino-5-((4-hydroxy-2-oxo-2H-chromen-3-yl) (3-methylphenyl) methyl)-1,3-dimethylpyrimidine-2,4(1H,3H)-dione (4j):

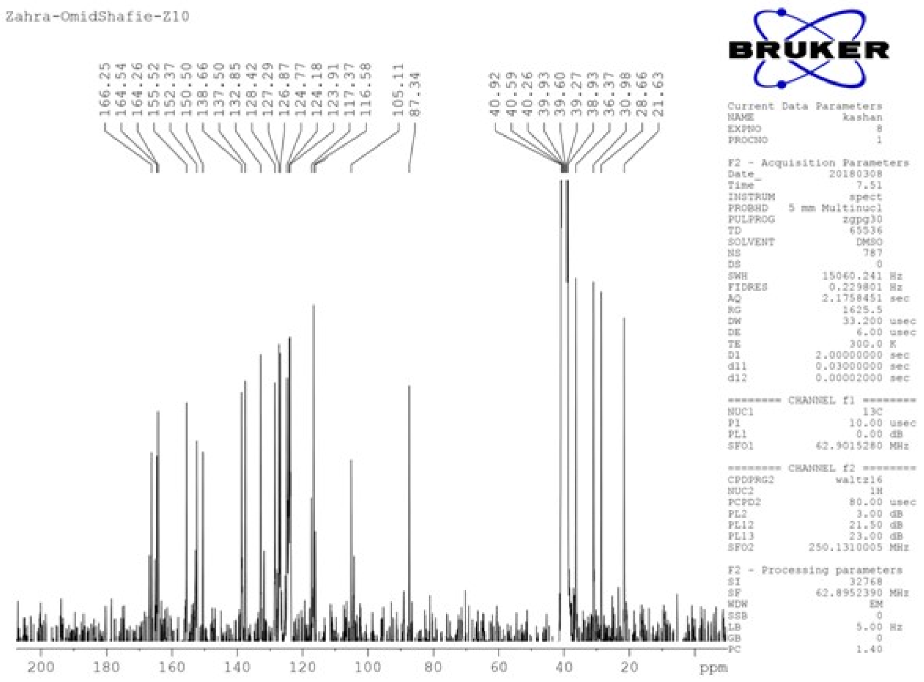

FT-IR: 6-Amino-5-((4-hydroxy-2-oxo-2H-chromen-3-yl) (4-bromophenyl) methyl)-1,3-dimethylpyrimidine-2,4(1H,3H)-dione (4k):

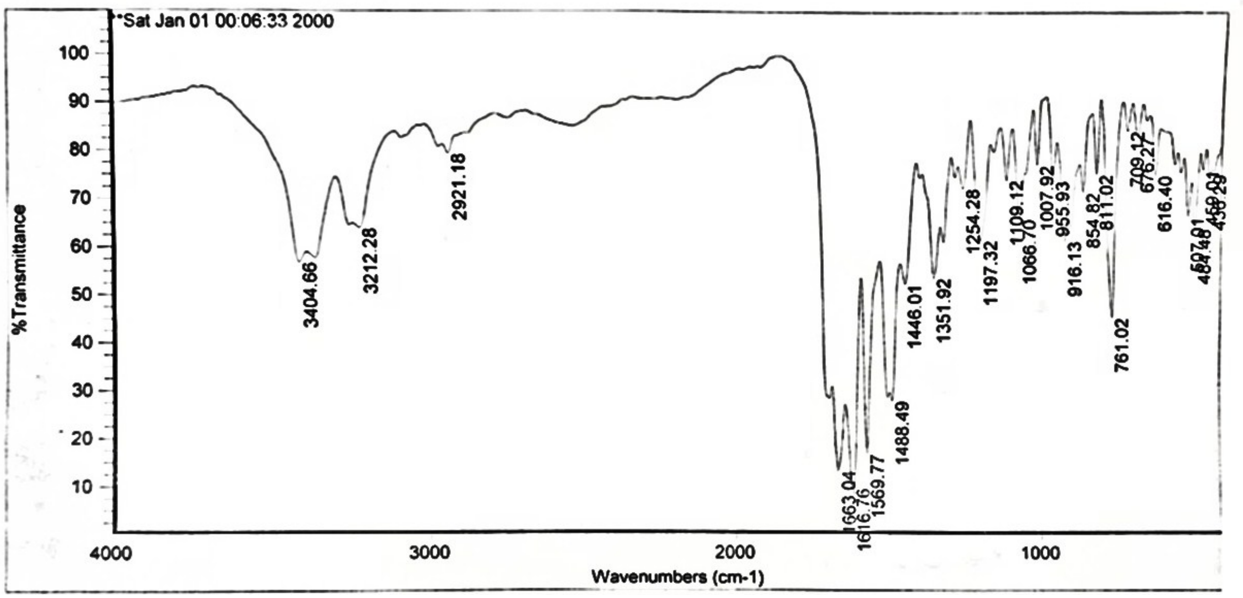

H NMR: 6-Amino-5-((4-hydroxy-2-oxo-2H-chromen-3-yl) (4-bromophenyl) methyl)-1,3-dimethylpyrimidine-2,4(1H,3H)-dione (4k):

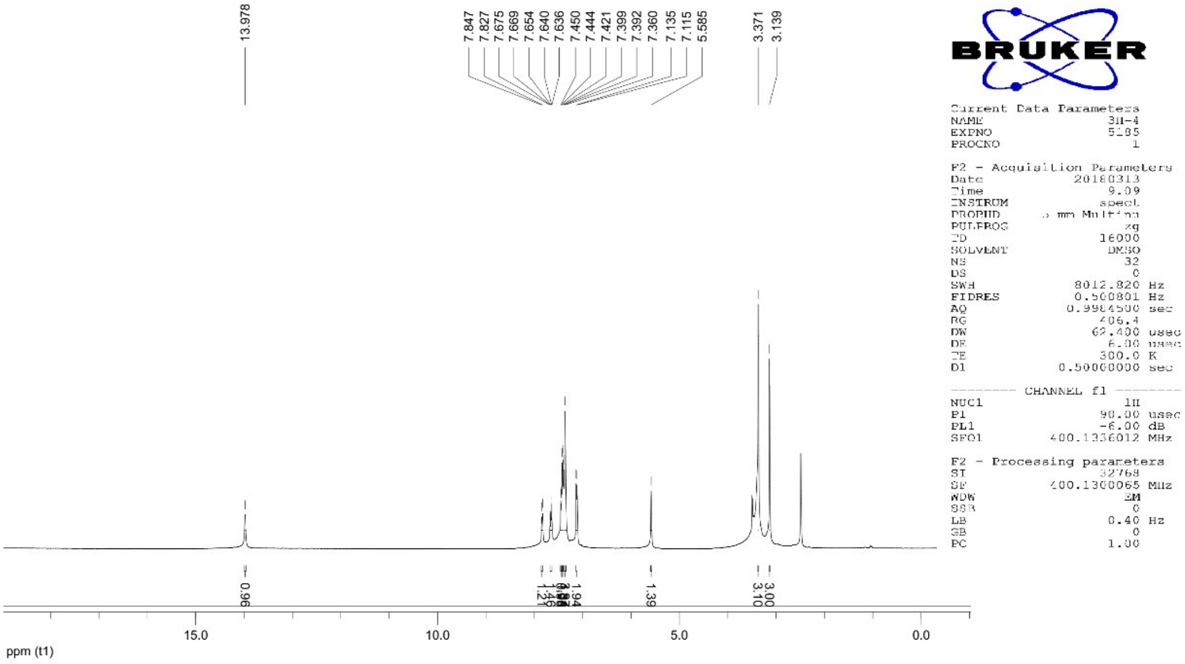

FT-IR: 6-Amino-5-((4-hydroxy-2-oxo-2H-chromen-3-yl) (4-isopropylphenyl) methyl)-1,3-dimethylpyrimidine-2,4(1H,3H)-dione (4l):

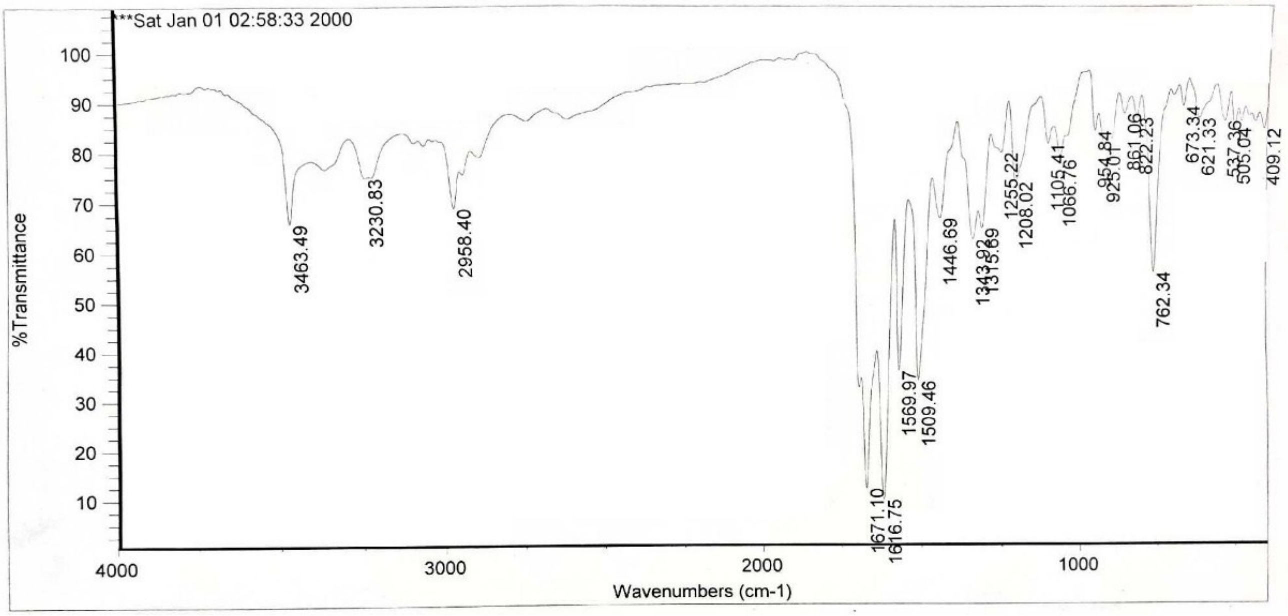

H NMR: 6-Amino-5-((4-hydroxy-2-oxo-2H-chromen-3-yl) (4-isopropylphenyl) methyl)-1,3-dimethylpyrimidine-2,4(1H,3H)-dione (4l):

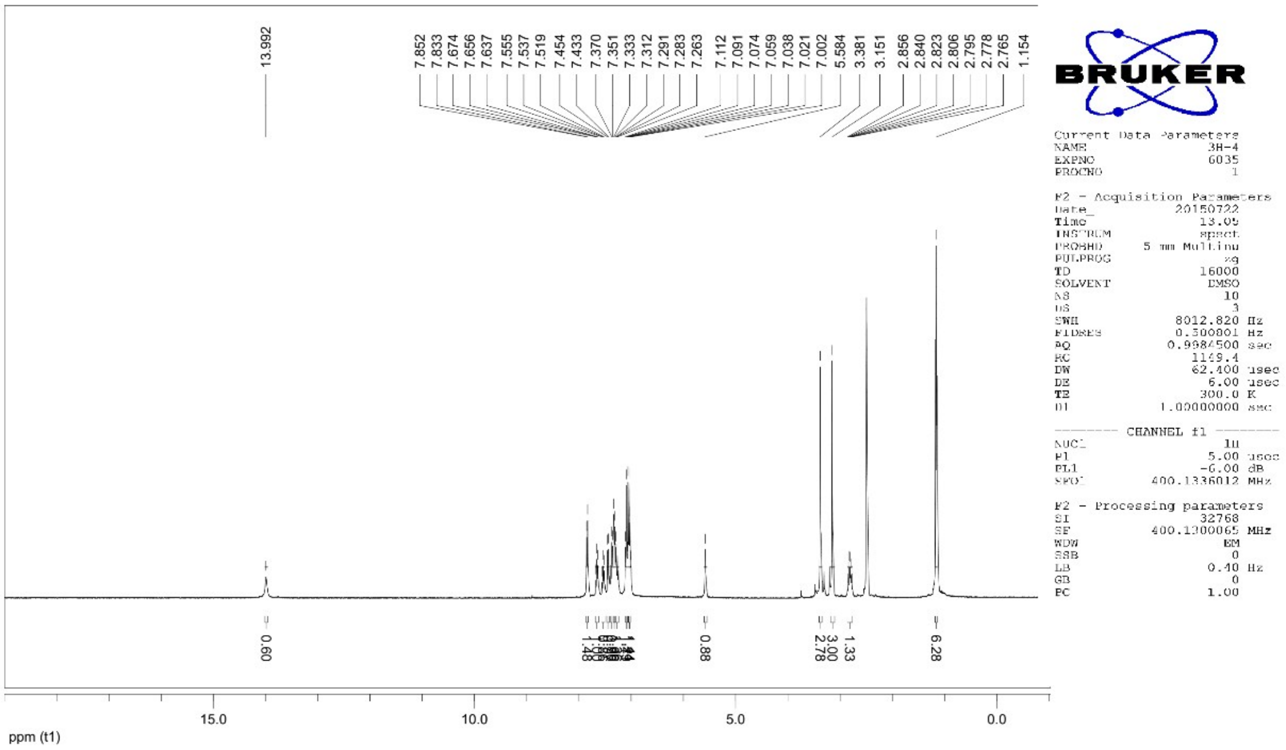

Supplement: RA-009-C9RA05896F-s001 [file RA-009-C9RA05896F-s001.pdf]
